# NCP1/AtMOB1A Plays Key Roles in Auxin-Mediated *Arabidopsis* Development

**DOI:** 10.1371/journal.pgen.1005923

**Published:** 2016-03-04

**Authors:** Xiaona Cui, Zhiai Guo, Lizhen Song, Yanli Wang, Youfa Cheng

**Affiliations:** 1 Key Laboratory of Plant Molecular Physiology, Institute of Botany, Chinese Academy of Sciences, Beijing, China; 2 University of Chinese Academy of Sciences, Beijing, China; Carnegie Institution for Science, UNITED STATES

## Abstract

MOB1 protein is a core component of the Hippo signaling pathway in animals where it is involved in controlling tissue growth and tumor suppression. Plant MOB1 proteins display high sequence homology to animal MOB1 proteins, but little is known regarding their role in plant growth and development. Herein we report the critical roles of *Arabidopsis MOB1* (*AtMOB1A*) in auxin-mediated development in *Arabidopsis*. We found that loss-of-function mutations in *AtMOB1A* completely eliminated the formation of cotyledons when combined with mutations in *PINOID* (*PID*), which encodes a Ser/Thr protein kinase that participates in auxin signaling and transport. We showed that *atmob1a* was fully rescued by its *Drosophila* counterpart, suggesting functional conservation. The *atmob1a pid* double mutants phenocopied several well-characterized mutant combinations that are defective in auxin biosynthesis or transport. Moreover, we demonstrated that *atmob1a* greatly enhanced several other known auxin mutants, suggesting that *AtMOB1A* plays a key role in auxin-mediated plant development. The *atmob1a* single mutant displayed defects in early embryogenesis and had shorter root and smaller flowers than wild type plants. *AtMOB1A* is uniformly expressed in embryos and suspensor cells during embryogenesis, consistent with its role in embryo development. AtMOB1A protein is localized to nucleus, cytoplasm, and associated to plasma membrane, suggesting that it plays roles in these subcellular localizations. Furthermore, we showed that disruption of *AtMOB1A* led to a reduced sensitivity to exogenous auxin. Our results demonstrated that *AtMOB1A* plays an important role in *Arabidopsis* development by promoting auxin signaling.

## Author Summary

MOB1 protein is a key component of the Hippo signaling pathway in animals, and it plays critical roles in organ size control. The plant hormone auxin regulates many aspects of plant growth and development including organogenesis. In this work, we showed that *AtMOB1A*, which is highly homologous to animal MOB1 proteins, plays an important role in plant organogenesis. Furthermore, we demonstrated that *AtMOB1A* synergistically interacts with auxin biosynthesis, transport, and signaling pathways to regulate *Arabidopsis* development. We further showed that *AtMOB1A* likely controls plant development by promoting auxin signaling. This work identified a new player in auxin-mediated plant development and lays a foundation for further dissection of the mechanisms by which auxin regulates organogenesis.

## Introduction

In recent years, the Hippo signaling pathway has emerged as a very important pathway for animal development [1]. This highly conserved pathway was initially identified in *Drosophila* as a key pathway controlling organ size, and later was shown to play a role in controlling cell fate and pattern formation in mammals [2–5]. The core part of the pathway is a phosphorylation cascade composed of four key components in mammals and *Drosophila*: a Ste20-like Ser/Thr protein kinase Mst1/2 [Hippo (Hpo) in *Drosophila*] [6,7], an NDR-family protein kinase Lats1/2 [Warts (Wts) in *Drosophila*] [8,9], and two kinase regulatory components, Sav and MOB1 (Sav and Mats in *Drosophila*) [10,11] (S1 Fig). Mst1/2 phosphorylates MOB1 and Lats1/2, and activates Lats1/2. MOB1 can bind to Lats1/2 and potentiate its intrinsic kinase activity. The activated Lats1/2 in turn phosphorylates and inactivates a transcriptional co-activator YAP/TAZ (Yorkie in *Drosophila*) [12]. YAP/TAZ is an effector of the Hippo pathway. Phosphorylation of YAP/TAZ results in its cytoplasmic retention, largely by facilitating its interaction with 14-3-3 proteins. Dephosphorylation of YAP/TAZ promotes its nuclear localization where it interacts with transcription factors and regulates gene expression. *Drosophila* mutants of core components in this pathway, such as *hpo*, *wts*, *mats*, *sav*, showed larger organs. In mammals, Hippo signaling controls patterning and differentiation of airway epithelial progenitors, mammary gland differentiation, intestinal fate, cardiovascular, liver, pancreas, central nervous system, and lymphocyte development [2]. It also regulates stem cell self-renewal and cell polarity in animals [2,13,14]. Recently, it was reported that the *Arabidopsis thaliana MOB1A* gene is required for tissue patterning of the root tip [15] and the development of both sporophyte and gametophyte [16]. MOB1 proteins in plants and animals share high sequence homology [11]. It is tempting to hypothesize that the Hippo pathway may also function in plants. However, very little is known regarding how the hypothesized Hippo pathway may regulate plant growth and development.

The plant hormone auxin plays critical roles in plant growth and development. Local auxin biosynthesis, polar transport, and auxin signaling all contribute to proper plant growth and development. The best characterized tryptophan-dependent auxin biosynthesis pathway is the indole-3-pyruvate pathway, in which tryptophan is converted into indole-3-pyruvate by TAA/TAR family of amino transferases. Indole-3-pyruvate is then converted into IAA by YUC family of flavin-containing monooxygenases [17–21]. Auxin biosynthesis is temporally and spatially regulated [22,23]. Auxin transport is carried out by auxin influx carriers AUX1/LAXs, auxin efflux carriers PINs, and ABCB transporters [24]. Both local auxin biosynthesis and polar transport are important for generating auxin gradients and maxima, which are perceived by auxin receptors. The best characterized auxin receptor is TIR1/AFBs and Aux/IAA co-receptor complexes [25,26]. Disruption of auxin biosynthesis, polar transport or signal transduction pathways leads to defects in almost every aspect of developmental processes, such as flower, embryo, root, and leaf development [22,27,28]. For example, auxin biosynthetic mutants *yuc1/4/10/11* quadruple mutants are defective in embryogenesis, and auxin signaling mutants such as *mp* fail to develop normal hypocotyls and roots [23]. Auxin transport mutant *pin1* develops pin-like inflorescences, which was also observed in auxin signaling mutant *mp* and *npy* mutants [29,30]. Although it has been well documented that auxin plays essential roles in plant development, little is actually understood regarding how auxin gradients are translated into guiding proper developmental events.

In this paper, we provide evidence that links *AtMOB1A*, which is homologous to a key component of the animal Hippo pathway, to auxin-mediated plant organogenesis and development. We conducted a genetic screen for mutants that could enhance the phenotypes of *pid*, which is defective in auxin signaling and transport [31,32]. One of the *pid* enhancers, *ncp1* (no-cotyledon in *pid 1*) failed to develop cotyledons in *pid* background. We further showed that *ncp1* single mutant displays strong developmental defects in early embryos, seedlings, and in adult plants. *NCP1* encodes a protein with significant homology to the animal MOB1s, a core component of the Hippo pathway. We showed that NCP1*/*AtMOB1A probably has biochemical activities similar to those of animal MOB1, because the *Drosophila MOB1* (*Mats*) can fully rescue the developmental defects of *ncp1/atmob1a*. The *atmob1a* mutant showed synergistic genetic interactions with known auxin biosynthetic, transport, and signaling mutants, suggesting that *AtMOB1A* functions in parallel to auxin pathways or affecting some aspects of auxin biology. Furthermore, disruption of *AtMOB1A* led to a decrease in sensitivity to auxin treatments and down-regulation of auxin reporters including *DR5-GFP*, *ProARF7*:*GUS*, and *ProARF19*:*GUS*. Our findings demonstrate that *AtMOB1A* likely promotes auxin signaling, thus impacting various *Arabidopsis* developmental processes.

## Results

### Identification of genetic enhancers of *pid*

**Fig 1:**
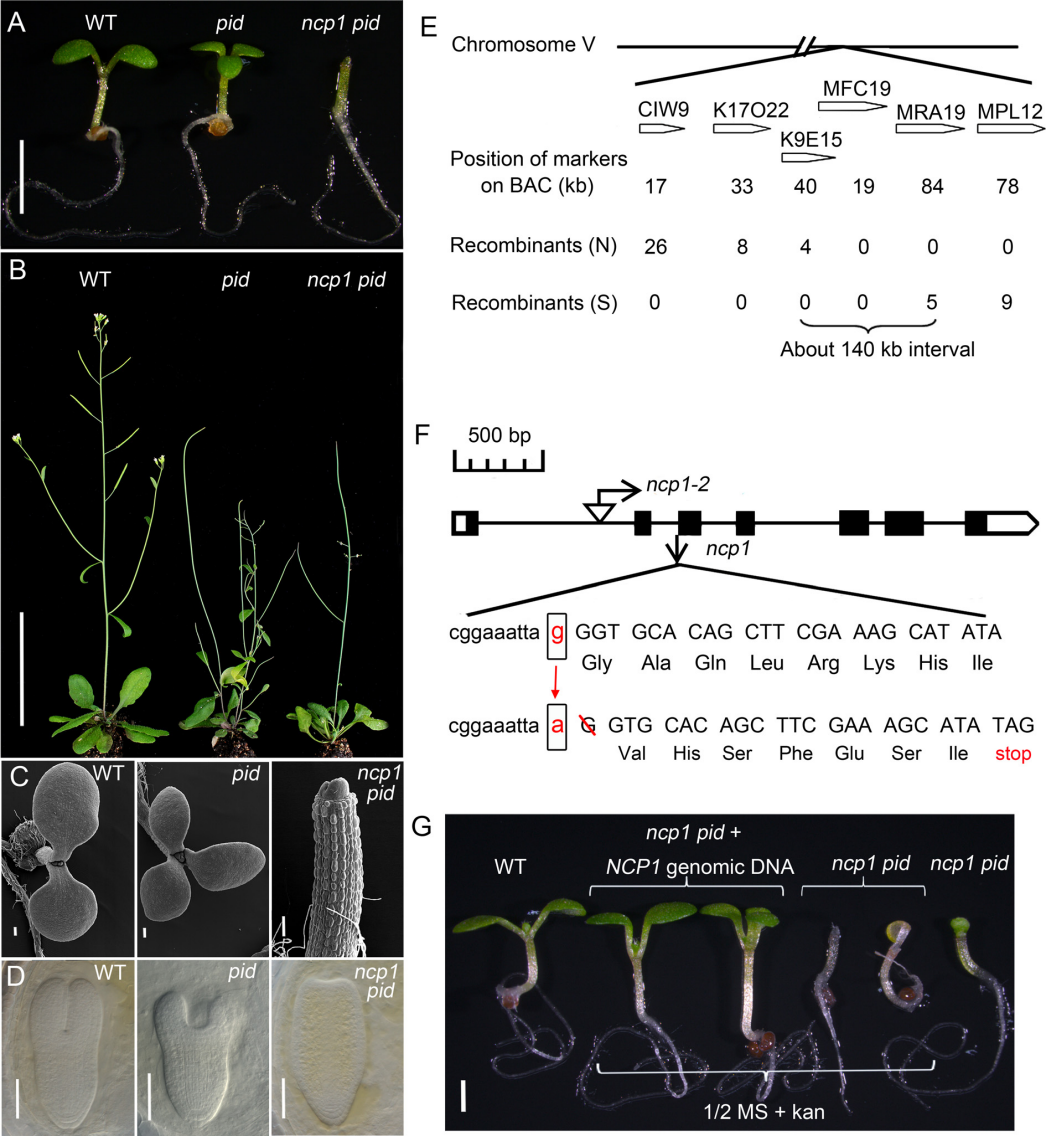
Identification and molecular cloning of *ncp1*. (A) Mutation in *NCP1* caused no-cotyledon phenotypes in seedling in *pid* background. From left to right, WT, *pid*, and *ncp1 pid*. (B) Adult plants of WT, *pid*, and *ncp1 pid*. (C) Electron micrographs of seedlings of WT (left), *pid* (middle), and *ncp1 pid* (right). Note that *ncp1 pid* failed to develop a cotyledon. (D) Late stage of embryos of WT, *pid*, and *ncp1 pid*. (E) Molecular cloning of *ncp1*. The mutation in *ncp1* was mapped to an interval of about 140 kb on Chromosome V. (F) A schematic gene structure of *At5g45550* and the location of T-DNA insertion of *ncp1-2*. Black boxes and lines designate exons and introns. The mutation at the splicing receptor of the second intron in *ncp1-1* led to a frame-shift and introduced a premature stop codon in cDNA. (G) Complementation of *ncp1-1* with a genomic DNA fragment of *At5g45550* gene. From left to right: WT and *ncp1 pid* with/without the *At5g45550* transgene (two seedlings of each). Note that the green transgenic seedlings had two or three cotyledons, while the yellowish non-transgenic seedlings had no cotyledon. The transformed *NCP1* gene restored *ncp1 pid* to *pid* phenotype. Scale bar, 2 mm (A), 5 cm (B), 100 μm (C), 25 μm (D), 1 mm (G).

Genetic enhancement has been widely used to identify components in signaling and metabolic pathways. We previously identified *npy1* as a genetic enhancer of *yuc1 yuc4*, which are defective in auxin biosynthesis. *NPY1* is a key component of a signaling pathway responsible for auxin-mediated organogenesis [29]. Previous studies have shown that several *Arabidopsis* auxin mutants/mutant combinations, including *npy1*, *yuc1 yuc4*, *wag1 wag2*, *pin1*, and *wei8 tar2*, have no cotyledons when combined with *pid*, which encodes a protein kinase important for auxin signaling and transport [20,29,30,33]. Therefore, *pid* provides a sensitized background, and cotyledon formation serves as an easy phenotypic readout for us to genetically identify additional components in auxin-mediated plant development. We conducted a genetic screen for enhancers of *pid* and isolated a new mutant that lacked cotyledons. We name the mutant as *ncp1* (*n**o-**c**otyledon in* *p**id 1*). At seedling stage, *ncp1 pid* failed to develop cotyledons, but they appeared to have normal hypocotyls and roots (Fig 1A and 1C). The no-cotyledon phenotype of *ncp1 pid* was highly penetrant: the majority (90%) of the mutants completely lacked both cotyledons, while some plants occasionally developed one cotyledon (Table 1). Interestingly, *ncp1 pid* plants could develop true leaves, however, they were abnormal in morphology and vascular development (S2 Fig). The *ncp1 pid* plants were able to transition from vegetative growth to reproductive development, but their inflorescences were all pin-like and failed to produce fertile flowers (Fig 1B). The no-cotyledon phenotype in seedlings of *ncp1 pid* was caused by defects occurred during embryogenesis. In mature embryos, the cotyledon formation was abolished in *ncp1 pid*, while two cotyledons in WT and two or three cotyledons developed in *pid* (Fig 1D).

**Table 1:** Genetic analysis of *ncp1 pid*.

		Mutant genotype analysis			Mutant phenotype analysis			
		No. of seedlings (% of total seedlings mutant seedlings)			No. of seedlings			
Parent genotype	Mutant genotype	expected	observed	Seedlings genotyped	with 2 cotyledons	with 3 cotyledons	no cotyledon	with 1 cotyledon
*pid* ^*+/-*^ *ncp1-1*^*-/-*^	*pid*^*+/+*^ *ncp1-1*^*-/-*^	61 (25)	75 (30)	244	75	0	0	0
*pid* ^*+/-*^ *ncp1-1*^*-/-*^	*pid* ^*+/-*^ *ncp1-1*^*-/-*^	122 (50)	105 (43)	244	103	2	0	0
*pid* ^*+/-*^ *ncp1-1*^*-/-*^	*pid* ^*-/-*^ *ncp1-1*^*-/-*^	61 (25)	64 (26)	244	0	0	58	6

The observed no-cotyledon phenotype was dependent on the presence of the *pid* mutation. We genotyped 48 individual plants that showed the no-cotyledon phenotype and found out that they were all *pid* homozygous, suggesting that the phenotype was dependent on the presence of the *pid* mutation. We further analyzed the progenies from a single *ncp1*^*+/-*^ *pid*^*+/-*^ plant, 22 of 427 seedlings (about 1/20) showed the no-cotyledon phenotype, indicating that the phenotype was caused by two un-linked recessive mutations, i.e. *pid* and *ncp1*.

We crossed *ncp1*^*+/-*^ *pid*^*+/-*^ to *Arabidopsis* Landsberg ecotype and allowed the F_1_ plants to self-fertilize to generate a mapping population. In the F_2_ mapping population, we isolated 1325 seedlings that failed to develop cotyledons from about 26,000 F_2_ individuals. We found that the no-cotyledon phenotype was linked to two genetic loci: one on the bottom arm of chromosome II and the other on chromosome V. The Chromosome II locus is *pid*, further supporting that the no-cotyledon phenotype was dependent on *pid*. We narrowed the mapping interval on Chromosome V down to about 140 kb, between the two genetic markers on K9E15 and MRA19 (Fig 1E). We sequenced all of the open reading frames (ORFs) in the mapping interval and identified a G to A conversion at the splicing junction of the second intron and the third exon of the gene *At5g45550*. Further analysis of *At5g45550* cDNA from the *ncp1* mutant plants revealed that the mutation caused a single base-pair shift of the splicing acceptor of the second intron and the deletion of the first G of the third exon. The mutation led to a frame shift after the Lys24, and introduced a premature stop codon (Fig 1F). Therefore, this mutant is likely a null allele.

To further confirm that the identified mutation in *At5g45550* was responsible for the observed no-cotyledon phenotype in *pid* background, we transformed a genomic fragment containing the coding region and its up- and down-stream regulatory sequences of *At5g45550* into *ncp1*^*-/-*^ *pid*^*+/-*^ plants. All of the T_1_ transgenic plants (341 in total) had two or three cotyledons. We genotyped the T_1_ plants and found that 86 of them were double mutants, indicating that wild type (WT) copy of *At5g45550* complemented the phenotype (Fig 1G).

We also identified a T-DNA insertion allele of *ncp1* (GK_719G04) from the NASC stock center, and named it *ncp1-2*. We generated double mutants *ncp1-2 pid* and *ncp1-2 pid-714* (SAIL_770_E05). Both of the double mutants displayed the same no-cotyledon phenotype as *ncp1-1 pid* (S3 Fig). Therefore, we conclude that *At5g45550* is *NCP1* and the identified mutations in *At5g45550* are responsible for the no-cotyledon phenotype in *pid* backgrounds. We used *ncp1-1* allele for further detailed analysis and genetic interaction studies in the paper.

### NCP1/AtMOB1A plays important roles in *Arabidopsis* development

*NCP1* was identified in the *pid* mutant background. We segregated out *pid* and investigated whether *ncp1-1* mutation alone caused any developmental defects. At seedling stage, *ncp1-1* had shorter root meristems zones when compared to WT. The root phenotypes of *ncp1-1* were caused by decreased cell numbers in its root meristem (S4E and S4F Fig). Compared to WT plants, *ncp1-1* single mutant plants were slightly taller with shorter siliques and smaller flowers (S4A–S4C Fig). The mutant was much less fertile. Our observed phenotypes of roots, siliques and flowers were consistent with previous findings from the analyses of the T-DNA allele of *AtMOB1A*, *GK_719G04* (*ncp1-2*) [15]. The shorter root phenotype and the decrease in cell numbers in the root meristems of *ncp1* and *ncp1 pid* may be caused by defects in cell division. To test this hypothesis, we investigated cell division activities in *ncp1* and *ncp1 pid* mutants. *CycB1;1*:*GUS* is a widely used marker for the G_2_/M phase of the cell cycle [34]. The GUS staining domains were dramatically decreased in both *ncp1* and *ncp1 pid* mutants, indicating that cell division activities were decreased in these mutants. These findings could partially account for the observed short root phenotypes of *ncp1* and *ncp1 pid* (S4I and S4J Fig).

**Fig 2:**
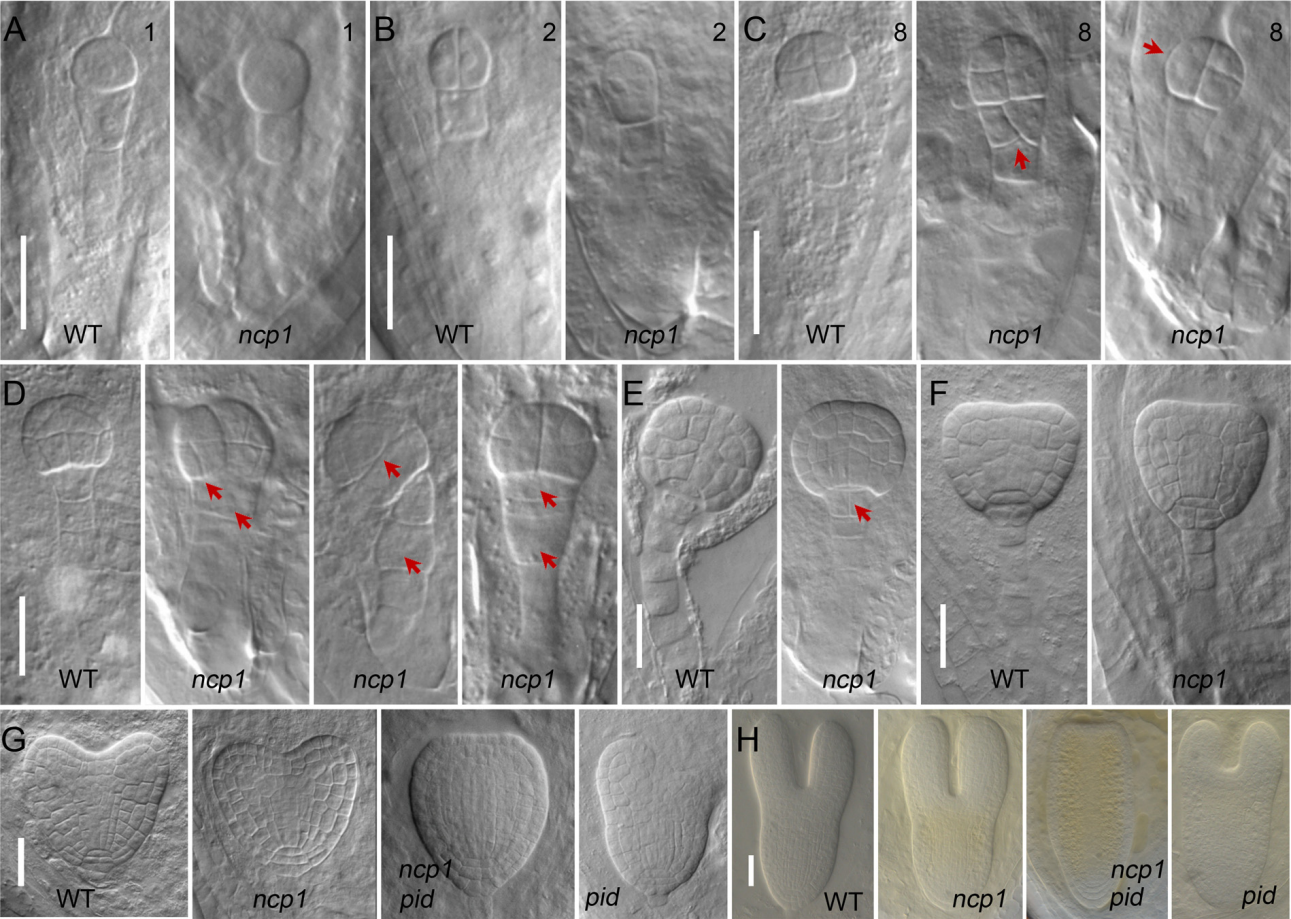
*NCP1* is important for embryogenesis. (A to F) Embryos of WT and *ncp1* from 1-cell stage to transition stage. Red arrowheads point to the abnormal cell divisions in pro-embryos and suspensor cells at 8-cell (C) and 16-cell stages in *ncp1-1* (D), and in hypophysis at the globular stage in *ncp1-1* (E). (G, H) Defects of *ncp1-1 pid* embryos at heart and torpedo stages. From left to right: WT, *ncp1-1*, *ncp1-1 pid*, and *pid*.

Because the no-cotyledon phenotype of *ncp1 pid* was caused by defects occurred during embryogenesis, we also analyzed whether disruption of *AtMOB1A* alone is sufficient to affect embryogenesis. Another indication that *AtMOB1A* is important for embryogenesis is that about 66.6% of the embryos (*n* = 785) were aborted in the *ncp1-1* siliques (S4D Fig). We carefully analyzed various stages of embryogenesis of the *ncp1-1* mutants and discovered that *AtMOB1A* plays an important role in early embryogenesis. The cell division in some mutant embryos was disturbed as early as 8-cell embryo stage. The upmost suspensor cell divided longitudinally in some *ncp1-1* embryos whereas the cell in WT divides horizontally (Fig 2C). At 16-cell stage, both the pro-embryos and suspensor cells were abnormal in *ncp1-1* (Fig 2D). At globular stage, the upmost suspensor cell became the hypophysis and remained as a lens-shape cell in WT [35]. But in *ncp1-1* mutant, it was no longer lens-shaped and was divided into 2 cells (Fig 2E). The observed defects in the embryogenesis of *ncp1-1* mutant would severely affect its embryo development, indicating that *NCP1* is important for embryogenesis.

### *NCP1* encodes a homolog of a key component in the animal Hippo signaling pathway

The predicted NCP1 protein contains 215 amino acid residues. It shares high sequence homology (63% identity) to the *Drosophila* MOB1 (Mats) (S5 Fig). MOB1 was first identified in yeast as Mps One Binder 1, an essential protein required for the completion of mitosis and maintenance of ploidy [36]. It has been shown that MOB1 is a key component of the Hippo signaling pathway [11]. In the *Arabidopsis* genome, there are four *MOB1*-like genes, *At5g45550*, *At4g19045*, *At5g20430*, and *At5g20440*. They have been renamed as *AtMOB1A*, *AtMOB1B* [15], *AtMOB1C*, and *AtMOB1D* herein, respectively.

MOB1 is highly conserved in plant species. For example, the MOB1As of *Brassica rapa* and *Arabis alpina* are almost identical to AtMOB1A: they differ from AtMOB1A in only one and three amino acid residues out of 215, respectively. Other putative plant MOB1A proteins and AtMOB1A share more than 90% identities (S5 Fig). It is clear from our phylogenetic analysis that animal MOB1 proteins and plant MOB1s belong to different clades (S6 Fig). The *MOB1* genes have duplicated in the most recent common ancestor of land plants (Embryophyte) during evolution, and evolved with frequent duplication or deletion in the derived lineages of land plants. *Selaginella moellendorffii* only has one *MOB1* gene whereas *Physcomitrella patens* has two. Monocots and dicots often have two to four copies of *MOB1* genes (S6 Fig).

**Fig 3:**
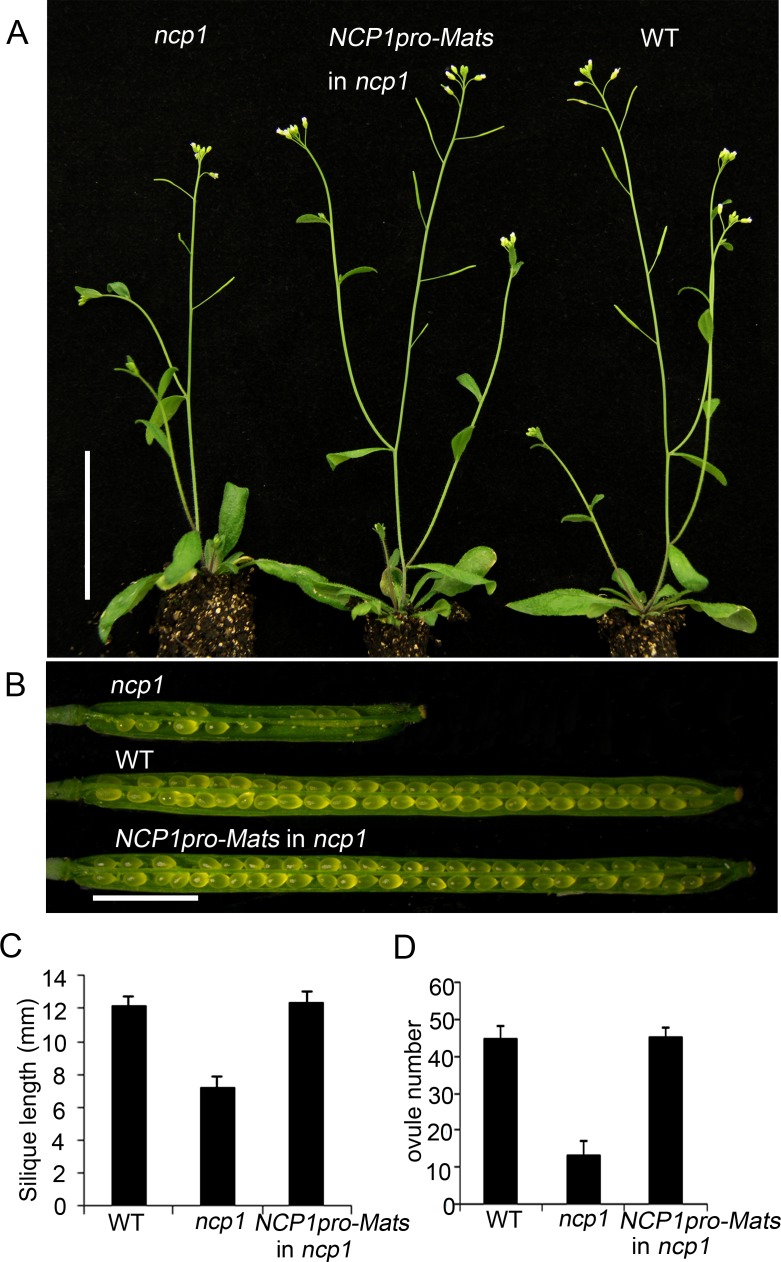
The *Drosophila Mats* gene can functionally substitute for *NCP1*. (A) From left to right: *ncp1*, *ncp1* with *Mats* under the control of the *NCP1* promoter, and WT. (B) Siliques of *ncp1*, WT, and *ncp1* with *Mats*. (C, D) Quantitative measurement of silique length (C) (*n* = 20), and ovule number per silique (D) (*n* = 10). Data are represented as mean ± SEM. Scale bar, 5 cm (A), 2 mm (B).

To further demonstrate that *NCP1* is functionally related to *MOB1* homologs from other organisms, we put the *Drosophila MOB1* (*Mats*) under the control of the *NCP1* promoter and transformed the construct into *ncp1-1*. We confirmed that all of the 88 transgenic *ncp1-1* seedlings contained the *Mats* gene. The adult plants of *ncp1-1* mutant showed severe defects in fertility, but the *Mats* transgenic *ncp1-1* mutant plants were able to produce siliques like WT (Fig 3). Our results indicated that the *Drosophila Mats* gene complemented the defects caused by *ncp1-1* mutation, and the function of MOB1/Mats is conserved from plants to *Drosophila*.

### NCP1/AtMOB1A is expressed during embryogenesis and is localized to several cellular compartments

**Fig 4:**
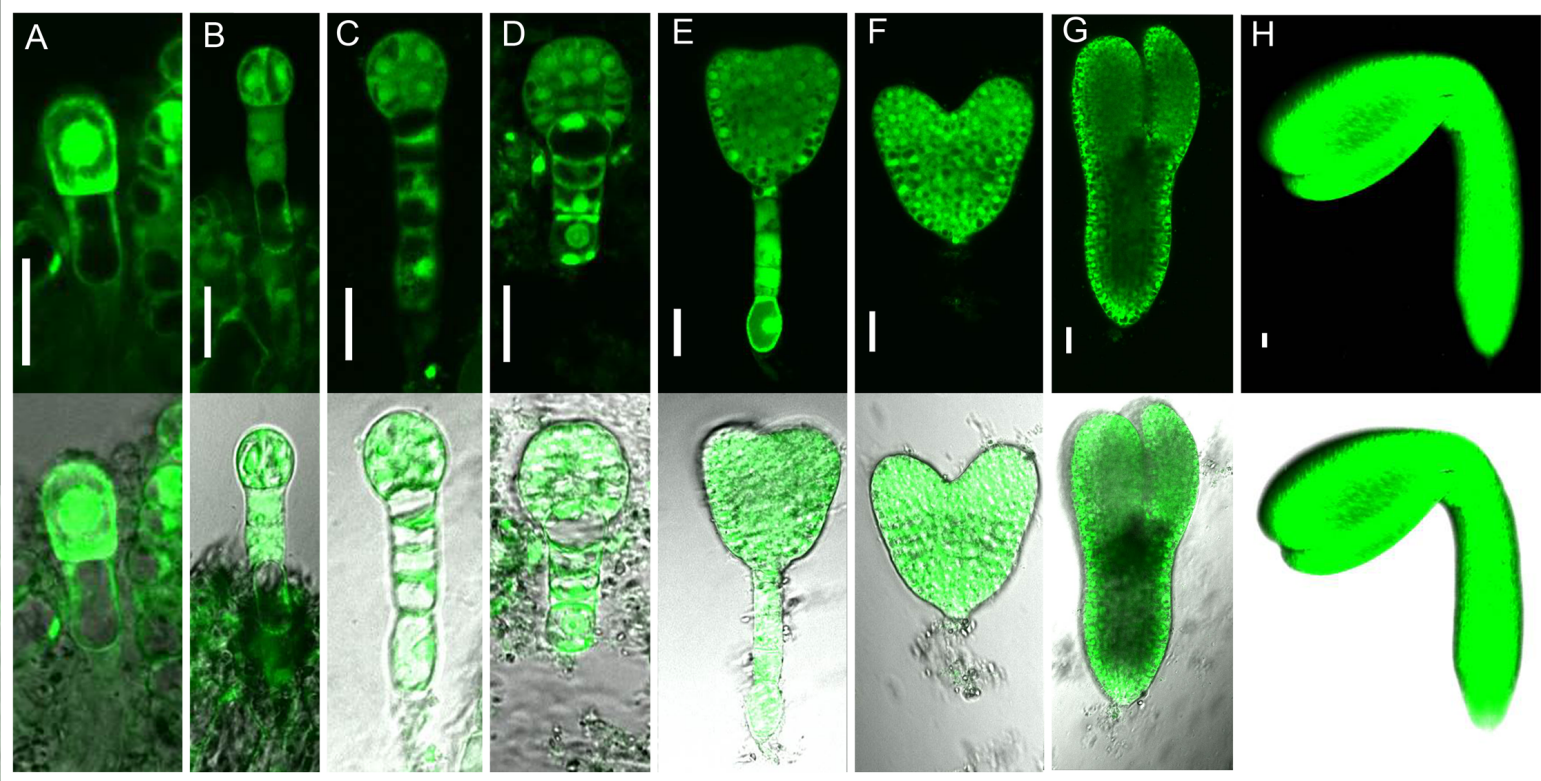
Expression patterns of AtMOB1A protein during embryogenesis. (A-H) Embryos from 1-cell stage to mature embryo stage. (A) 1-cell embryo. (B) 2-cell embryo. (C) 4–8 cell embryo. (D) Globular embryo. (E) Transition stage embryo. (F) Heart stage embryo. (G) Torpedo stage embryo. (H) Cotyledon stage embryo. Note that AtMOB1A protein is localized to nucleus, cytoplasm, and associated to plasma membrane. Scale bar, 20 μm.

To investigate the expression pattern of *NCP1/AtMOB1A*, we generated a construct containing the *NCP1* genomic DNA including its regulatory and coding sequences, with the *GFP* gene inserted immediately before the stop codon. We transformed *ncp1* and *ncp1 pid*^*+/-*^ mutants with this construct and found that the construct complemented both *ncp1* and *ncp1 pid*, indicating that the NCP1-GFP fusion protein was fully functional. *AtMOB1A* is uniformly expressed in embryonic and suspensor cells from one-cell to mature embryo stages. The expression patterns of *AtMOB1A* are consistent with its role in embryo development. AtMOB1A protein is localized to nucleus, cytoplasm and associated to plasma membrane (Fig 4). The observed nuclear localization was consistent with previously findings [16,37].

### Synergistic genetic interaction between *ncp1/atmob1a* and various auxin mutants

*The ncp1-1* mutant was isolated as an enhancer of *pid*, which is a well-known auxin mutant. We further analyzed whether *ncp1* could genetically interact with other known auxin mutants. We tested three groups of auxin mutants that are defective in either auxin biosynthesis, or transport, or signaling.

**Fig 5:**
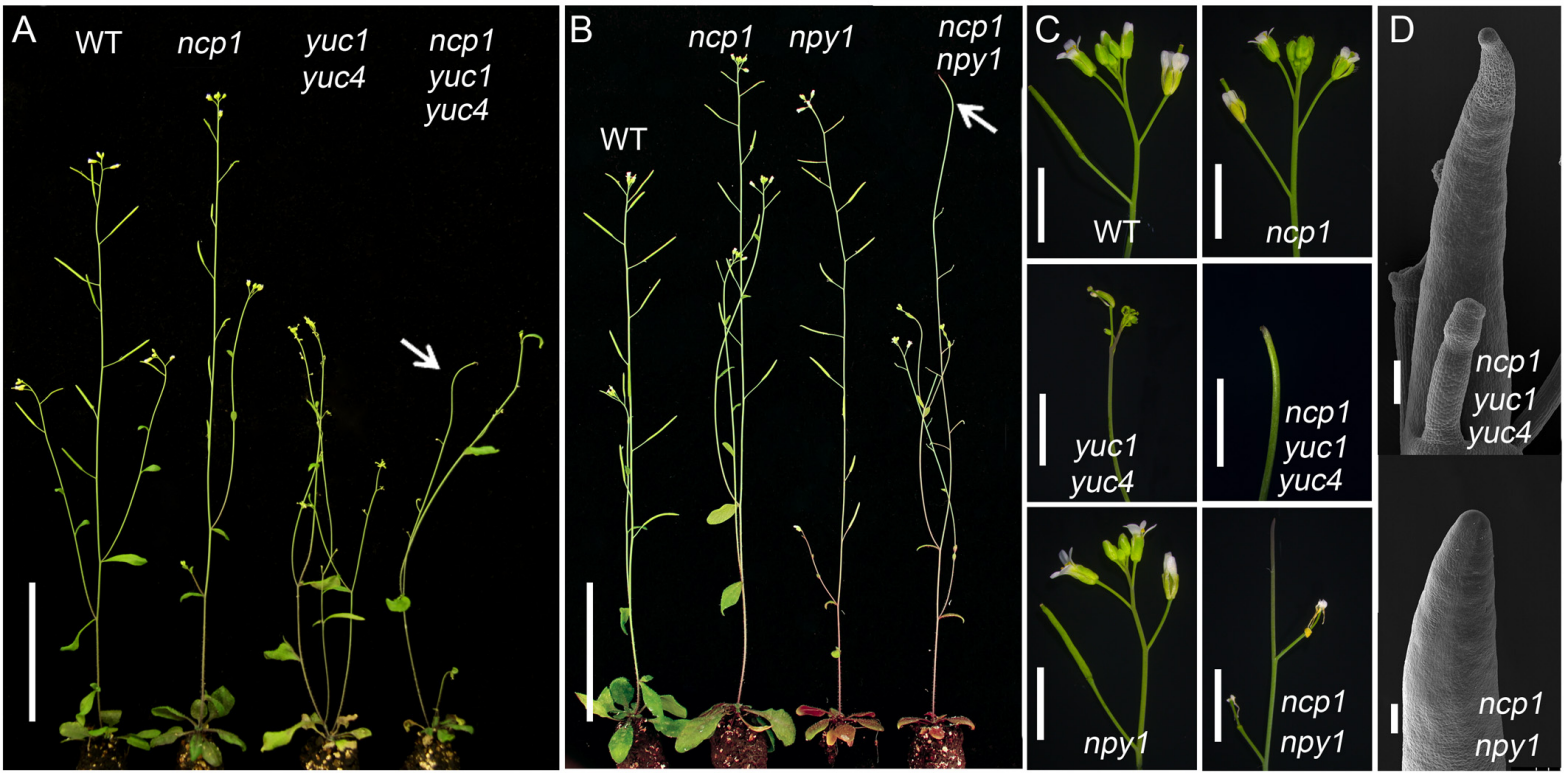
Genetic interactions between *ncp1* and mutants of auxin biosynthesis and polar transport. (A) *ncp1* enhanced *yuc1 yuc4* mutants phenotypes. (B) *ncp1* enhanced *npy1* mutant phenotypes. Arrowheads point to the pin-like inflorescence in *ncp1 yuc1 yuc4* (A) and *ncp1 npy1* (B). (C) Inflorescences of WT, *ncp1*, *yuc1 yuc4*, *ncp1 yuc1 yuc4*, *npy1*, *ncp1 npy1*. (D) SEM micrographs of representative pin-like inflorescences in *ncp1 yuc1 yuc4* and *ncp1 npy1* mutants. Scale bars, 5 cm (A and B), 5 mm (C), 100 μm (D).

It has been shown that YUC flavin-containing monooxygenases and TAA1/TAR tryptophan amino transferases define a main auxin biosynthetic pathway in *Arabidopsis* [19,20]. Both YUCs and TAAs play essential roles in all of the major developmental processes including embryogenesis and flower development in *Arabidopsis* [18,22,23]. When we disrupted *NCP1* in *yuc1 yuc4* background, the resulting triple mutants developed pin-like inflorescence whereas *yuc1 yuc4* never form pins, demonstrating that *ncp1* greatly enhanced the phenotypes of auxin biosynthetic mutants (Fig 5A and 5C and 5D).

We previously reported that NPY1 is involved in auxin-mediated organogenesis. The *pid npy1* double mutants had no cotyledons, and *npy1 yuc1 yuc4* triple mutants developed pin-like inflorescences. NPY1 is proposed to play a role in auxin transport and signaling [29,30]. When we introduced *ncp1* into *npy1* background, the double mutants produced pin-like structures whereas either single mutant did not form any pins (Fig 5B and 5C and 5D).

**Fig 6:**
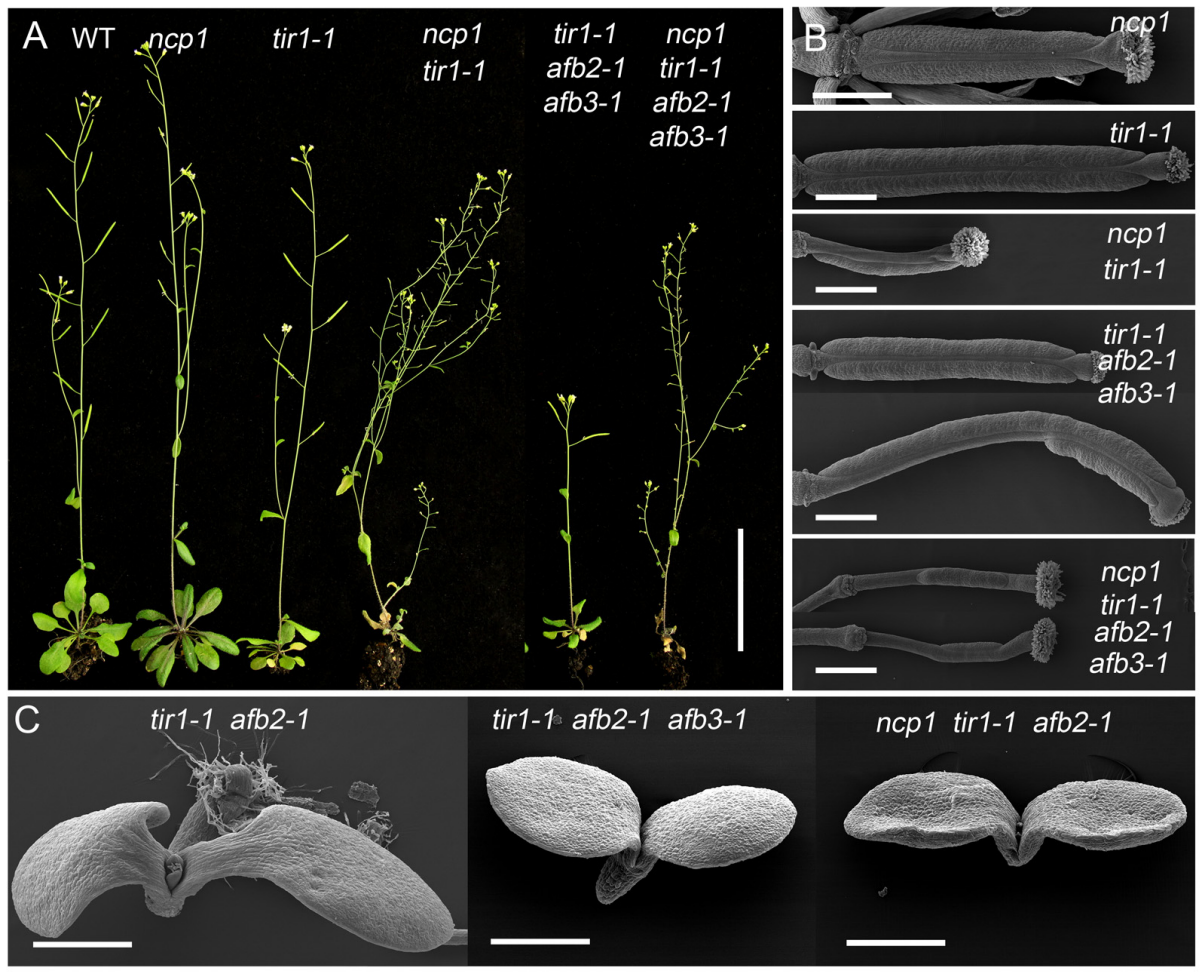
Genetic interactions between *ncp1* and auxin signaling mutants. (A) From left to right: adult plants of WT, *ncp1*, *tir1-1*, *ncp1 tir1-1*, *tir1-1 afb2-1 afb3-1*, *ncp1 tir1-1 afb2-1 afb3-1*. Note the decreased fertility of *ncp1 tir1-1* and completely sterile phenotypes of *ncp1 tir1-1 afb2-1 afb3-1*. (B) From top to bottom: gynoecia of *tir1-1*, *ncp1 tir1-1*, *tir1-1 afb2-1 afb3-1*, *ncp1 tir1-1 afb2-1 afb3-1*. (C) From left to right: Seedlings of *tir1-1 afb2-1*, *tir1-1 afb2-1 afb3-1*, *ncp1 tir1-1 afb2-1 afb3-1*. Scale bars, 5 cm (A), 500 μm (B and C).

We next tested if *ncp1* could synergistically interact with auxin signaling mutants. TIR1/AFBs are the best characterized auxin receptors responsible for regulating expression of auxin inducible genes. We crossed *ncp1* to *tir1-1 afb2-1 afb3-1* [27] and obtained various combinations of *ncp1* and *tir1 afb* mutants from the F_2_ populations. The phenotypic analysis was performed in F_4_ generation. The single mutants of *tir1-1*, *afb2-1*, *afb3-1*, and combinations of their double mutants did not display dramatic developmental defects under normal growth conditions [27]. Interestingly, the *ncp1 tir1-1* double mutants showed severe reduction in fertility, which was caused mainly by the defects in gynoecium patterning. The defects were further enhanced in *ncp1 tir1-1 afb2-1 afb3-1* and led to complete sterility (Fig 6A and 6B). The adult plants of *tir1-1 afb2-1* double mutants showed a reduction in rosette leaf size and inflorescence height, but their seedlings were similar to WT (Fig 6C) [27]. However, the *ncp1 tir1-1 afb2-1* triple mutants exhibited strong developmental defects. Six of 34 (18%) triple homozygous seedlings of *ncp1 tir1-1 afb2-1* mutants had no roots, whereas the *tir1-1 afb2-1* double mutants never displayed such phenotypes. The observed no-root phenotypes closely resembled those of *bdl/iaa12* or *mp/arf5* mutants. The *tir1-1 afb2-1 afb3-1* and *tir1-1 afb1-1 afb2-1 afb3-1* mutants also showed the *mp*-like rootless seedling phenotypes at frequency of 36% and 49%, respectively (Fig 6C) [27]. Our results indicated that *ncp1* genetically interacts with auxin signaling pathway.

In *Arabidopsis*, *PID* has three close homologs: *WAG1*, *WAG2*, and *PID2*, which redundantly control cotyledon development [30]. The *ncp1 pid* double mutants phenocopied the *pid wag1 wag2 pid2* quadruple mutants (S7A and S7B Fig). We further tested if *ncp1* could enhance *pid wag1 wag2 pid2* phenotypes. The double or higher orders of mutant combinations between *ncp1* and *wag1 wag2 pid2* did not show obvious phenotypic enhancement, suggesting that *PID* played a more predominant role in regulating cotyledon development than *WAG1*, *WAG2*, *and PID2*. The *ncp1 pid wag1 wag2 pid2* quintuple mutants displayed no-cotyledon phenotype similar to that of *pid wag1 wag2 pid2*. However, the quintuple mutants showed strong developmental defects in true leaves (S7 Fig). In dark grown seedlings, the initiation of true leaves was delayed in the quintuple mutants, compared to *ncp1 pid* (S7C Fig). In 14-day-old light grown seedlings, the quintuple mutants developed single or two leaves, and occasionally developed a pin-like true leaf (S7D and S7E Fig). In 36-day-old plants, the quintuple mutants showed two types of phenotypes. The type I plants (44%, *n* = 61) developed one to three true leaves and a pin-like inflorescence, and were arrested at this developmental stage. The type II plants (56%, *n* = 61) could produce more than three true leaves, and continued to grow with the phenotypes similar to those of *ncp1 pid* (S7F Fig).

We showed that *NCP1* genetically interacted with *PID* to control cotyledon development in *Arabidopsis*. In animals, MOB1 physically interacts with and activates NDR/LATS through recruitment to the plasma membrane [38,39]. Because both PID and NDR/LATS are AGC kinases, we hypothesized that AtMOB1A may use a mechanism analogous to that of animal MOB1. PID may play a role equivalent to that of NDR/LATS. To test this hypothesis, we conducted both pull-down and Co-IP assays to determine whether AtMOB1A physically interacts with PID/WAGs. However, we did not detect direct physical interactions between NCP1/AtMOB1A and PID, or WAG1/2 in our experiments (S8 Fig). These results suggested that there may not be direct interactions between NCP1 and PID/WAGs, or the interactions are transient and difficult to be detected under our assay conditions. There are at least 39 AGCs in Arabidopsis. The observed genetic synergism of AtMOB1A with PID may suggest that AtMOB1A is necessary for the function of other AGCs that have overlapping functions with PID/WAG1/WAG2.

### Auxin responses are decreased in *ncp1* and *ncp1 pid* mutants

To assess the role of *NCP1* in auxin response, we introduced the auxin reporter DR5-GFP into *ncp1*, *pid*, and *ncp1 pid* mutants background. At heart and torpedo stages of embryogenesis, strong DR5-GFP signals were observed at the cotyledon primordia and hypophysis in WT. In *pid*, the DR5-GFP signals remained similar to WT. In contrast, the GFP signals were significantly decreased at the cotyledon primordia in *ncp1* single and *ncp1 pid* double mutants. It is worth noting that the auxin responses at hypophysis seemed not changed in *ncp1* single and *ncp1 pid* double mutants (S9A Fig). These observations suggested that *NCP1* might be involved in auxin signaling.

It is known that auxin is required for the initiation and growth of lateral root (LR) and root hairs, and exogenous auxin can stimulate these developmental processes [40]. To further investigate the roles of *NCP1* in auxin responses, we examined the response of *ncp1* mutant to exogenous auxin treatment. Four-day-old seedlings of WT, *pid*, *ncp1*, and *ncp1 pid* germinated on 1/2 strength of Murashige and Skoog medium (MS) plates were transferred and grew on 1/2 MS plates containing 50 nM 2,4-D, a synthetic auxin. It is obvious that the lengths of root hairs and density of LR/LR primordium were dramatically increased in WT, *pid* and *ncp1*, however the effects of exogenous auxin on *ncp1 pid* were much weaker (S9B–S9F Fig). This suggested that *ncp1 pid* double mutants are partially resistant to auxin in terms of root hair and LR initiation and growth. The pericycle cells in *ncp1* and *ncp1 pid* were similar to WT, suggesting that the LR defects in *ncp1 pid* was likely due to slow LR primordium growth and the failure to emerge from the epidermis of the primary root. It could also be a defect in pre-branch site formation, which is not morphologically distinct.

*ARF7* and *ARF19* redundantly control LR development, and they are expressed in lateral and/or primary roots [41,42]. We analyzed the expression of *ARF7* and *ARF19* in seedlings of *ncp1 pid* mutants by using *ProARF7*:*GUS* and *ProARF19*:*GUS* reporter lines [41,42]. The expression levels of *ProARF7*:*GUS* and *ProARF19*:*GUS* were dramatically decreased in the LR primordium of *ncp1*, *pid*, and *ncp1 pid* mutants, compared to WT. The expression levels of *ProARF19*:*GUS* were also reduced in primary roots of *ncp1*, *pid*, and *ncp1 pid* mutants (S10 Fig). These findings suggested that the LR defects in *ncp1 pid* were partially caused by down-regulation of *ARF7* and *ARF19*.

### The expression pattern but not its sub-cellular localization of PIN1-GFP is altered in *ncp1 pid* double mutants during embryogenesis

PIN1 plays an important role during embryogenesis [28]. It is reported that PIN1-GFP is asymmetrically localized on plasma membrane [43,44]. We introduced the PIN1-GFP marker into *ncp1*, *pid*, and *ncp1 pid* mutants, and carefully checked the subcellular localization of PIN1-GFP from transition to torpedo stage of embryogenesis. No obvious alteration of the subcellular localization of PIN1-GFP was observed in these stages. However, we found that the expression levels of PIN1-GFP were altered in *ncp1* mutants compared to WT. At these stages, PIN1-GFP was mainly expressed at the cotyledon primordia and ground tissue, which formed a Y-shape pattern. At transition stage, the expression pattern of PIN1-GFP in *ncp1* mutants was similar to that of WT. However, in *ncp1 pid* double mutants, PIN1-GFP was found to be mainly expressed at the epidermal cell layer of apical part of embryos and ground tissue, which were barely connected by weak PIN1-GFP-expressing cells (S11 Fig). This result suggested that *NCP1* plays a role in controlling the expression pattern of *PIN1*. It was previously reported that the localization pattern of PIN1 appeared normal in roots of *Mob1A RNAi* seedlings [15]. The discrepancy between our findings and those of the previous study might be because of the tissue specificity.

## Discussion

The Hippo signaling pathway has been shown to play a critical role in organ size control and morphogenesis in animals, but it is still an open question whether the Hippo pathway exists in plants. Because MOB1 proteins share high sequence homology in animals and plants, it is tempting to hypothesize that the Hippo pathway may also exist and play a role in plant growth and development. Here we show that *AtMOB1A* is functionally conserved with the *Drosophila* protein because *atmob1a* was fully rescued by its *Drosophila* counterpart, suggesting that at least part of the Hippo pathway is functional in plants. *NCP1/AtMOB1A* synergistically interacts with key genes in auxin biosynthesis, transport, and signal transduction pathways to regulate *Arabidopsis* development. The observed synergistic genetic interactions and the decreased auxin responses in various *ncp1* and auxin mutant combinations suggest that there is an intrinsic link between auxin pathway and the hypothesized Hippo pathway in plants. Our finding that the expression levels of *ProARF7*:*GUS* and *ProARF19*:*GUS* were dramatically decreased in *ncp1 pid* further supports the notion that *AtMOB1A* is important for auxin-mediated developmental processes. This work provides a genetic framework for the Hippo pathway in auxin-mediated plant development.

It was reported that about 2% of the progeny of *AtMob1A* RNAi silenced plants were tetraploid [16], which is a result of cell division defects. Auxin is also known to control plant development by regulating cell division and expansion. Therefore, *AtMOB1A* may be involved in auxin-controlled cell division. The mutants in animal Hippo pathway display defects in organ overgrowth [1], due to a loss of control of cell proliferation. In *ncp1* mutant, the length and the cell number of root meristem were decreased compared to WT (S4 Fig). The different developmental outcomes between animals and plant *mob1* mutants suggest that Hippo pathway/MOB1 protein may play different roles in plants and animals regarding cell proliferation. Recently, the Hippo pathway has been shown to control cell fate in animals. For example, the Hippo pathway activity is essential for the maintenance of the differentiated hepatocyte state. Acute inactivation of the Hippo signaling *in vivo* is sufficient to dedifferentiate adult hepatocytes into cells bearing progenitor characteristics [4]. In *Arabidopsis*, *ncp1 yuc yuc4* and *ncp1 npy1* mutants failed to develop flowers (Fig 5). The cotyledons were also eliminated in *ncp1 pid*, and the hypophysis was lost in *ncp1 tir1-1 afb2-1* during embryogenesis (Fig 2 and Fig 6). The observed defects in organ and embryo development in these mutants indicated that the Hippo pathway also plays a critical role in determining cell fate in plants.

It has been shown that the Hippo pathway is highly conserved in mammals and insects. A human *MOB1* gene rescued the developmental defects of the *Drosophila MOB1* mutant *mats* [11]. We show that the *Drosophila Mats* fully rescued developmental defects of the *Arabidopsis ncp1* mutant (Fig 3), indicating that at least some of the components of the Hippo pathway are conserved between plants and animals. This functional conservation of MOB1 proteins is consistent with the high similarities of their amino acid sequences (S5 Fig). It has been shown that MOB1 is a phospho-protein in animal systems. Phosphorylation of Thr12 and Thr35 of hMOB1 by MST1 or MST2 is required for the interaction of hMOB1 with NDR/LATS kinases in human [45,46]. Thr12 and Thr35 are absolutely conserved in MOB1s of plants and animals (S5 Fig). Both AtMOB1A and AtMOB1B were identified as phospho-proteins in a proteomic study [47], suggesting AtMOB1A/B is also phosphorylated by some kinase(s). AtMOB1A may also interact with *Arabidopsis* NDR/LATS kinases. In line with this hypothesis, there are eight NDR-like kinase genes in *Arabidopsis* [48], and they share high similarities with their human counterparts (S12 Fig).

It is well known that auxin promotes root hair and LR formation [40]. Gain-of-function mutant *msg2* of *Aux/IAA19* had severely reduced LR and LR formation was not normally induced by exogenous auxin [49]. Root hair and LR formation are also inhibited in *arf7 arf19* double mutants [41,42]. *pid* did not show obvious defects in root development [31]. However, *NCP1* and *PID* synergistically control LR formation and root hair growth in seedlings (S9 Fig). *ncp1 pid* also displayed strong defects in LR development in response to exogenous auxin treatment (S9 Fig). Expression levels of *ProARF7*:*GUS* and *ProARF19*:*GUS* were decreased in *ncp1 pid*. Moreover, *ncp1* enhanced *tir1-1 afb2-1* mutants’ phenotypes (Fig 6). These findings suggested that *NCP1/AtMOB1A* plays a positive role in promoting auxin signaling.

In the Hippo pathway, MOB1 binds and activates the AGC kinase NDR/LATS1/2 [38,39]. In *Arabidopsis*, there are 39 AGC kinases [48]. Some of them have been demonstrated to be involved to auxin pathways, such as PID/WAGs and D6PKs [31,50,51], which phosphorylate PIN1 at different phosphosites with different preference [52]. *d6pk0123* quadruple mutants showed somewhat pin-like axillary shoots [51]. *pid wag1 wag2* mutants phenocopied *ncp1 pid* [30]. Because *NCP1/AtMOB1A* is functionally conserved *MOB1* in *Arabidopsis*, it is possible that PID/WAGs/D6PKs function as a plant counterpart of LATS1/2. It would be interesting to test if PID/WAGs/D6PKs can rescue *Drosophila lats* mutant phenotypes. AtMOB1A may associate with PID/WAGs/D6PKs and regulates its kinase activity, which subsequently modifies activities of PIN1. In human and *Drosophila*, MOB1 can activate LATS/NDRs when targeted to the plasma membrane [39,53]. AtMOB1A is localized to nucleus [16,37] and also associated with plasma membrane (Fig 4). PID, WAGs and D6PKs are also associated with plasma membrane [54,55], making it possible for AtMOB1A to activate PID/WAGs/D6PKs. However, we did not detect direct physical interactions between AtMOB1A and PID/WAGs by using pull-down and Co-IP assays. The negative results do not rule out the possibility that AtMOB1A is in a complex with AGC kinases. On the other hand, *ncp1 pid wag1 wag2 pid2* showed no-cotyledon phenotypes similar to those of *pid wag1 wag2 pid2*. But the quintuple mutants displayed enhanced developmental defects in true leaves. These findings support the hypothesis that AtMOB1A may function with PID/WAGs. Alternatively, AtMOB1A and PID/WAGs/D6PKs may regulate transcription levels of auxin related genes. Indeed, we observed the alteration of expression pattern of PIN1-GFP and down-regulation of *ARF7*:*GUS* and *ARF19*:*GUS* in *ncp1 pid* double mutants (S10 Fig and S11 Fig). This finding is consistent with the mechanism that the animal Hippo pathway functions through regulating expression of downstream genes via a common growth regulatory effector, the transcriptional co-activator YAP/TAZ [1]. Another possibility is that the Hippo pathway functions in parallel to auxin pathway, yet they crosstalk to control plant development. This would be similar to the crosstalk between Wnt/β-catenin pathway and Hippo pathway to regulate animal development and tumorigenesis. It has been shown that cytoplasmic TAZ of the Hippo pathway can bind to DVL of the Wnt/β-catenin pathway and negatively regulate the Wnt/β-catenin pathway [56].

In conclusion, we demonstrate that *AtMOB1A*, a key component of the Hippo pathway, plays critical roles in auxin-mediated development in *Arabidopsis*. *AtMOB1A* synergistically interacts with auxin biosynthesis, transport, and signaling pathways to regulate *Arabidopsis* development. MOB1 is a regulator of AGC kinases in animal systems. PID/WAGs, D6PKs are AGC kinases, suggesting that NCP1/AtMOB1A may also regulate kinase activities of PID/WAGs and D6PKs, and possibly other AGC kinases in *Arabidopsis*. The fact that auxin responses and expression of auxin related genes such as *ARF7* and *ARF19* were down-regulated in *ncp1 pid* mutants suggests that *NCP1/AtMOB1A* may promote auxin signaling. This provides another layer of regulation of plant development by auxin. Further identification of other components of the Hippo pathway in *Arabidopsis* will help elucidate the mechanisms.

## Materials and Methods

### Plant materials and growth conditions

Plants were grown under 16-h light/8-h dark cycle at 22℃. The T-DNA insertion lines were obtained from NASC. The mutants used in this work were: *pid* (SALK_049736), *pid-714* (SAIL_770_E05), *ncp1-2* (GK_719G04). T-DNA insertion sites were determined by sequencing. Genotyping primers for *pid* (SALK_049736) and *pid-714* (SAIL_770_E05) are: 5’-CCTCAGATTTCGCTTACGCAG-3’, and 5’- GCGAGACGAGTGAATCGTCG-3’, combined with JMLB1 and SAIL-LB1, respectively. For genotyping *ncp1-2* (GK_719G04), 5’-ATGGATTCGTGTGGCTTTC-3’, 5’-TGTTTACAGCAAGCCATTC-3’, and PGABI1: 5’-ATATTGACCATCATACTCATTGC-3’ were used. To genotype *ncp1-1*, 5’-TGACCGTCTTCTTCCTAT-3’ and 5’-TGTTTACAGCAAGCCATTC-3’ were used and the PCR products were digested with *Mse*I. *npy1-2*, *yuc1*, *yuc4*, *tir1-1*, *afb2-1*, *afb3-1* were previously described [22,27,29]. All T-DNA insertion lines were genotyped as described previously [57–59].

### Constructs and transgenic plants

For complementation of *ncp1 pid* mutants, a genomic DNA fragment containing the coding region as well as up- and down-stream regulatory sequences of *At5g45550* was amplified by PCR using the following primers: 5’-CCCCCCGGGGAAACGGTGACCAAAATGCT-3’ and 5’-GCTCTAGAAGACGAGGCTCCAACACG-3’. The PCR product was digested with *BamH*I and *Xba*I and subcloned into *pPZP211* vector [60] to generate *pPZP211-NCP1gDNA*. The plasmid was transformed into *ncp1 pid*^*+/-*^ mutants via Agrobacterium strain GV3101 using floral dipping method [61]. The transgenic seedlings were selected on 1/2 MS plates containing 50 μg/mL kanamycin.

For expression of the *Drosophila Mats* under the control of *NCP1* promoter, the *Mats* cDNA was amplified with PCR using the primers: 5’-ACTCCCGGGATGGACTTCTTGTTCGGTTC-3’, and 5’- GCTCTAGACTATATCTGCCGCTCATCCT-3’. The *NCP1* promoter was amplified with primers: 5’-ACTGTCGACCTGCCCAATCAGCAAGAA-3’ and 5’-ACTCCCGGGGGCGACAAAAAGCAAGCGAG-3’. The PCR products were digested with *Sal*I, *Xma*I and *Xba*I and subcloned into *pCambia-1300* to generate *pCambia-1300-NCP1p*:*Mats*.

For expression pattern and subcellular analysis of NCP1, the *pPZP211-NCP1gDNA* construct was modified. The *GFP* gene was inserted immediate before the stop codon of *NCP1* gene with restriction site of *Apa*I.

### Microscopic analysis

SEM samples were prepared as described previously [62], and analyzed using a HITACHI S-4800 FESEM microscope. For whole-mount analysis of vascular structures and embryos, samples were prepared as previously described [63], and photographed under differential interference contrast (DIC) field or dark field on Leica DM 4500 and Leica S8AP0 microscopes. DR5-GFP and PIN1-GFP signals in embryos were viewed on Olympus FV1000MPE following the manufacturer’s instructions.

### Phylogenetic analysis

Sequences were aligned using Clustal X version 1.81 [64], then refined manually. Maximum Likelihood method was used to reconstruct the phylogenetic tree using Mega5 [65]. Topological robustness of the phylogenetic tree was assessed by bootstrapping with 1000 replicates [66].

### Pull-down and Co-IP assays

For the pull-down assay, cDNA of *PID* and *NCP1* was cloned into *pGEX-4T-1* and *pET30a* vectors to generate the expression constructs. The His-tagged and GST-tagged proteins were expressed in *E*. *coli* strain BL21. The subsequent protein purification and pull-down assay with Glutathione Sepharose 4B (GE) or His beads (Bio-Rad Ni-NTA Agarose) were carried out following the manufacturers’ manuals. The bound proteins were eluted and analyzed with anti-GST and anti-HIS antibodies (CWBIO).

To perform Co-IP assay of NCP1 and PID/WAGs, we constructed *pEarleyGate104-35S*:*YFP-NCP1*, *pSuper1300*:*PID-Myc*, *pSuper1300*:*WAG1-Myc*, *pSuper1300*:*WAG2-Myc*. *YFP-NCP1* and *PID-Myc* or *WAGs-Myc* constructs were transformed into tobacco (*Nicotiana Benthamiana*) by injection. Leaves were grounded into fine powder in liquid nitrogen. Proteins were extracted with the extraction buffer [100 mM HEPES (pH 7.5), 5 mM EDTA, 5 mM EGTA, 10 mM NaF, 5% Glycerol, 10 mM Na_3_VO_4_, 10 mM DTT, 1 mM PMSF, 0.1% Triton X-100, 10 μg/mL Aprotinin, 10 μg/mL Leupeptin, 10 μg/mL Antipain]. The protein extracts were spun twice for 30 min at 14,000 g at 4℃. The supernatant was incubated for 3 hr with anti-Myc-tag mAb-agarose (MBL) in IP buffer [20 mM Tris-HCl (pH 7.5), 150 mM NaCl, 1 mM EDTA, 1 mM EGTA, 1 mM Na_3_VO_4_, 1 mM NaF, 10 mM glycerophosphate, 0.1% Triton X-100, 1 μg/mL Aprotinin, 1 μg/mL Leupeptin, 1 μg/mL Antipain]. The agarose was washed for three times with 1 ml of PBS. Proteins were then released and collected by boiling in 2×SDS loading buffer for 5 min. IP products were detected by SDS-PAGE and immunoblot analysis using anti-Myc or anti-GFP antibodies (CWBIO). These experiments were repeated at least three times.

## Supporting Information

S1 Fig Schematic model of the Hippo signaling pathway. The Hippo pathway is highly conserved between *Drosophila* and mammals. Shown here is the core part of the pathway in mammals: a Ste20-like Ser/Thr protein kinase Mst1/2, an NDR-family protein kinase Lats1/2, and two kinase regulatory components, Sav and MOB1. Mst1/2 phosphorylates MOB1 and Lats1/2, and activates Lats1/2. MOB1 can bind to Lats1/2 and potentiate its intrinsic kinase activity. The activated Lats1/2 phosphorylates and inactivates the transcriptional co-activator YAP/TAZ. Dephosphorylation of YAP/TAZ promotes its nuclear localization where it interacts with transcription factors and regulates gene expression.
(TIF)

S2 Fig Diverse leaf phenotypes and vascular tissue defects of *ncp1 pid* young plants. (A) Various morphological phenotypes of true leaves in *ncp1 pid* mutants. Note the cup-shaped first true leaf in the up-left plant. (B-E) Venation patterns in leaves of WT (B), *ncp1* (C), *pid* (D), and *ncp1 pid* (E). Note the parallel venation in *ncp1 pid* (E). Scale bar, 1 mm.
(TIF)

S3 Fig Allelic analysis of *ncp1 pid*. (A) Additional combinations of *ncp1 pid* double mutants. From left to right: WT, *pid*, *ncp1-2*, *ncp1-2 pid*, *pid-714*, and *ncp1-2 pid-714*. Note the no-cotyledon phenotype of *ncp1-2 pid* and *ncp1-2 pid-714* seedlings. (B) Close-up of *ncp1-2 pid* and *ncp1-2 pid-714* seedlings. Note the trichomes on the true leaves of the double mutants. Scale bar, 5 mm (A), 500 μm (B).
(TIF)

S4 Fig Various developmental defects in flower and root of *ncp1* and *ncp1 pid*. (A) The *ncp1* plant is slightly taller than the WT plant. (B-E) Flowers of *ncp1* are smaller (B, C) and siliques (D) are shorter with some aborted seeds, and root meristems (E) of *ncp1* and *ncp1 pid* are shorter than WT. (F-H) Quantitative measurements of root length (F), root meristem region length (G), and root meristem cell number (H) (*n* = 20). (I) *CycB1;1*:*GUS* expression at 5 days after germination (DAG). (J) Quantification of *CycB1;1*:*GUS* spots (*n* = 10). Data are represented as mean ± SEM. Scale bar, 5 cm (A), 2 mm (D), 100 μm (E).
(TIF)

S5 Fig Sequence alignment of MOB1 proteins. MOB1 protein sequences of representative plant and animal species are aligned. Dicotyledons: *Arabidopsis thaliana*, *Brassica rapa*, *Solanum lycopersicum*, *Cucumis sativus*, *Populus trichocarpa*, *Glycine max*. Monocotyledons: *Oryza sativa*, *Hordeum Vulgare*, *Saccharum hybrid*. Lycophyte: *Selaginella moellendorffii*. Moss: *Physcomitrella patens*. Mammal: *Homo sapiens*. Insect: *Drosophila melanogaster*. Percentage of identity and NCBI accession numbers are listed at the end of each sequence. Residues that are identical in all sequences aligned are highlighted in black, and similar residues are in grey. The Thr12 and Thr35 residues are labeled with asterisks.
(TIF)

S6 Fig Phylogenetic relationships of MOB1 proteins from representative species of vertebrate, invertebrate, dicotyledons, monocotyledons, lycophyte, moss, and fungi. NCBI accession numbers are listed after the names of species.
(TIF)

S7 Fig Genetic interactions between *ncp1* and *pid wag1 wag2 pid2* mutants. (A) Light grown seedlings at 5 DAG. (B) Close-up view of mutant seedlings in (A). (C) SEM micrograph of dark grown seedlings at 3 DAG. Note the top of the seedlings. (D) Seedlings at 14 DAG. (E) Close-up view of true leaf development in *ncp1 pid wag1 wag2 pid2* mutants at 14 DAG. (F) Plants at 36 DAG. Note the type I and type II phenotypes of *ncp1 pid wag1 wag2 pid2* mutants. Scale bars, 1 mm (A, D), 500 μm (B, E), 100 μm (C), 1 cm (F).
(TIF)

S8 Fig Pull-down and Co-IP assays of NCP1 and PID/WAGs. (A, B) Pull-down assay with His (A) and GST tags (B). (C-E) Co-IP assay with YFP and Myc tags.
(TIF)

S9 Fig Auxin responses were reduced in *ncp1* and *ncp1 pid*. (A) DR5-GFP auxin response reporter in late heart stage embryos of WT, *ncp1*, *pid* and *ncp1 pid*. Note the arrowheads point to cotyledon primordia, where DR5-GFP signal was reduced in *ncp1* and *ncp1 pid* mutants. (B) Root hair initiation and elongation of WT, *ncp1*, *pid* and *ncp1 pid* seedlings in response to exogenous auxin treatment. 4-day-old seedlings grown on 1/2 MS plate were transferred onto plates containing 0 nM (mock, top panel) or 50 nM 2,4-D (bottom panel) and grew for 4 days. (C) Lateral root initiation of WT, *ncp1*, *pid* and *ncp1 pid* seedlings in response to exogenous auxin treatment. 4-day-old seedlings grown on 1/2 MS plate were transferred onto plates containing 0 nM (mock, top panel) or 50 nM 2,4-D (bottom panel) and grew for 4 days. (D-F) Quantitative measurements of LR density (number of emerged LR per portion of the primary root where LRs are present, D), LR primordium density (number of LR primordium per portion of the primary root where LR primordia are present, E), and LR initiation density (number of non-emerged LR primordia and emerged LRs per portion of the primary root, F) of seedlings in response to exogenous auxin treatment. 4-day-old seedlings grown on 1/2 MS plate were transferred onto plates containing 0 nM (mock) or 50 nM 2,4-D and grew for 4 days. Data are represented as mean ± SEM. Scale bar, 20 μm (A), 500 μm (B), 1 cm (C).
(TIF)

S10 Fig *ProARF7*:*GUS* and *Pro ARF19*:*GUS* expression levels were decreased in lateral root of *ncp1 pid* mutants. 10-d old seedlings were used for GUS staining. (A) *ProARF19*:*GUS*, from left to right: seedlings, primary roots, and lateral roots. (B) *ProARF7*:*GUS*, from left to right: seedlings and lateral roots. Scale bar, 1 mm (seedlings), 200 μm (primary roots), 50 μm (lateral roots).
(TIF)

S11 Fig Expression patterns of PIN1-GFP were altered in *ncp1* and *ncp1 pid* embryos. Heart (A) and late heart (B) stages of WT, *ncp1*, *pid* and *ncp1 pid*. Scale bar, 20 μm (A and B). Note the arrowheads point to the regions between cotyledon primordium and ground tissue, where PIN1-GFP was expressed in WT embryos but barely in mutants.
(TIF)

S12 Fig Sequence alignment of NDR/LATS proteins. NDR/LATS protein sequences of *Arabidopsis* and human are aligned.
(TIF)
