# Supplementary figures and images for: NCP1/AtMOB1A Plays Key Roles in Auxin-Mediated Arabidopsis Development
Source: PLoS Genet. 2016 Mar 4;12(3):e1005923. doi: 10.1371/journal.pgen.1005923 (PMC4778850; doi:10.1371/journal.pgen.1005923)

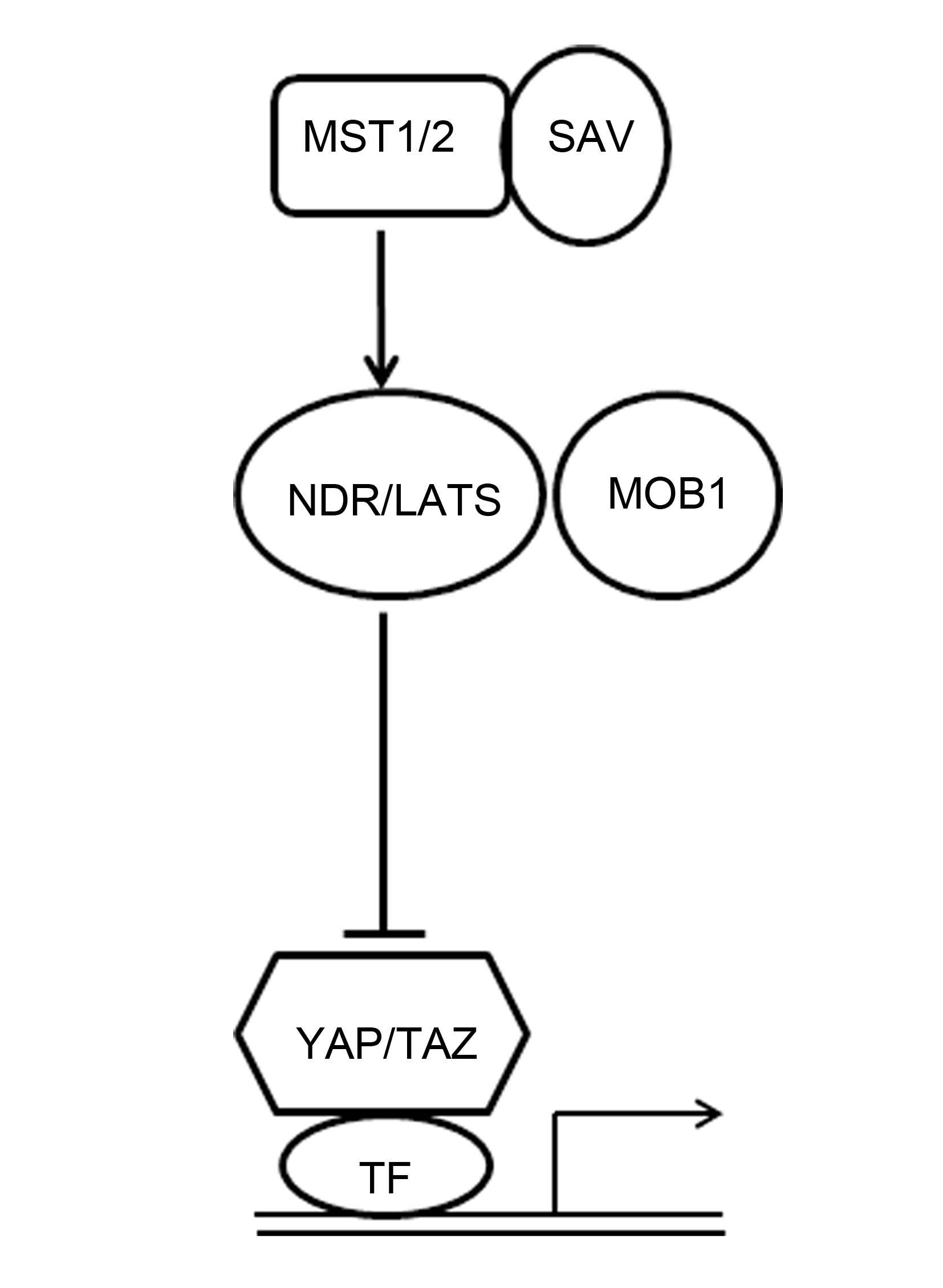

Supplement: S1 Fig — The Hippo pathway is highly conserved between Drosophila and mammals. Shown here is the core part of the pathway in mammals: a Ste20-like Ser/Thr protein kinase Mst1/2, an NDR-family protein kinase Lats1/2, and two kinase regulatory components, Sav and MOB1. Mst1/2 phosphorylates MOB1 and Lats1/2, and activates Lats1/2. MOB1 can bind to Lats1/2 and potentiate its intrinsic kinase activity. The activated Lats1/2 phosphorylates and inactivates the transcriptional co-activator YAP/TAZ. Dephosphorylation of YAP/TAZ promotes its nuclear localization where it interacts with transcription factors and regulates gene expression. (TIF) [file pgen.1005923.s001.tif]

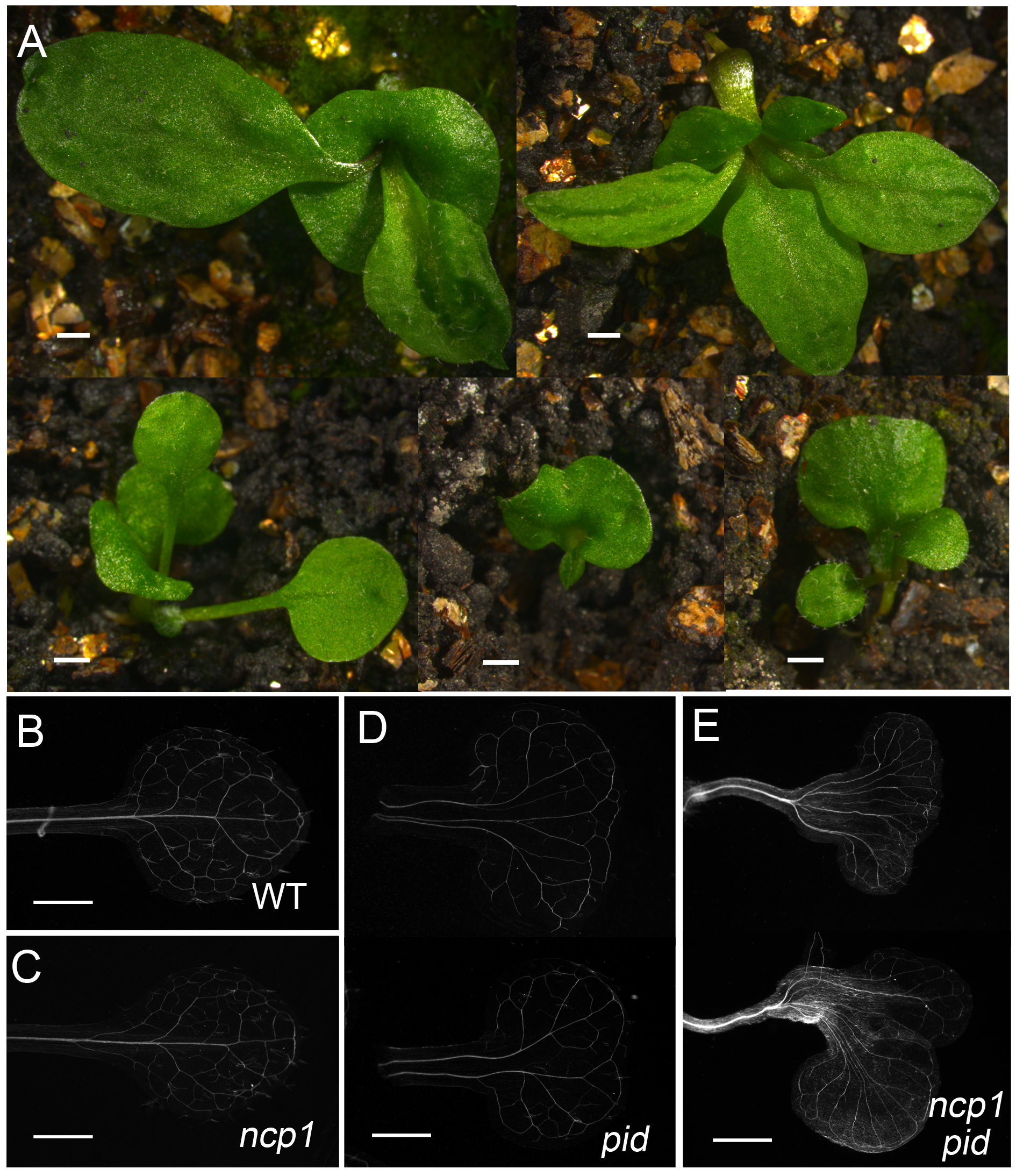

Supplement: S2 Fig — (A) Various morphological phenotypes of true leaves in ncp1 pid mutants. Note the cup-shaped first true leaf in the up-left plant. (B-E) Venation patterns in leaves of WT (B), ncp1 (C), pid (D), and ncp1 pid (E). Note the parallel venation in ncp1 pid (E). Scale bar, 1 mm. (TIF) [file pgen.1005923.s002.tif]

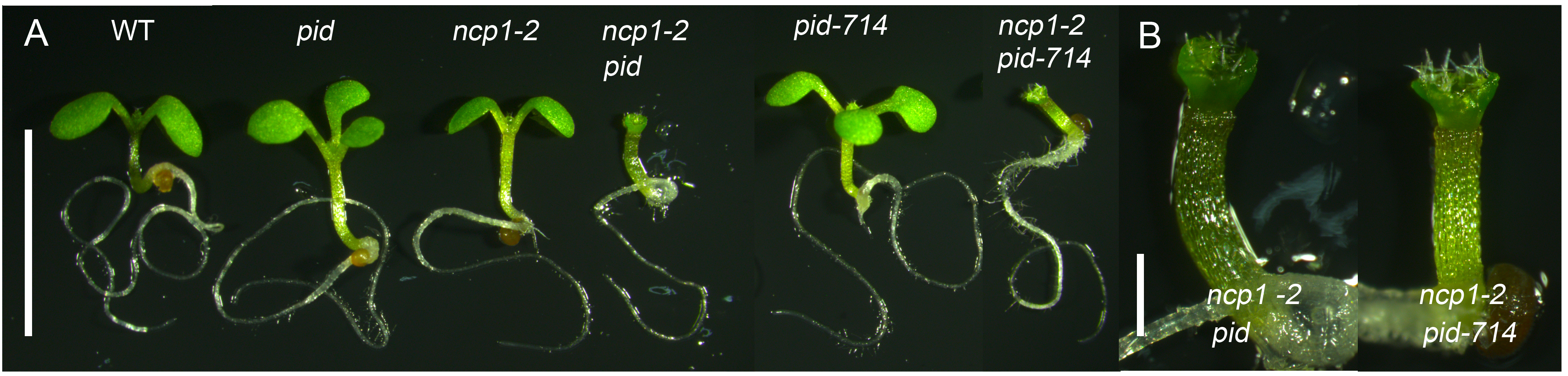

Supplement: S3 Fig — (A) Additional combinations of ncp1 pid double mutants. From left to right: WT, pid, ncp1-2, ncp1-2 pid, pid-714, and ncp1-2 pid-714. Note the no-cotyledon phenotype of ncp1-2 pid and ncp1-2 pid-714 seedlings. (B) Close-up of ncp1-2 pid and ncp1-2 pid-714 seedlings. Note the trichomes on the true leaves of the double mutants. Scale bar, 5 mm (A), 500 μm (B). (TIF) [file pgen.1005923.s003.tif]

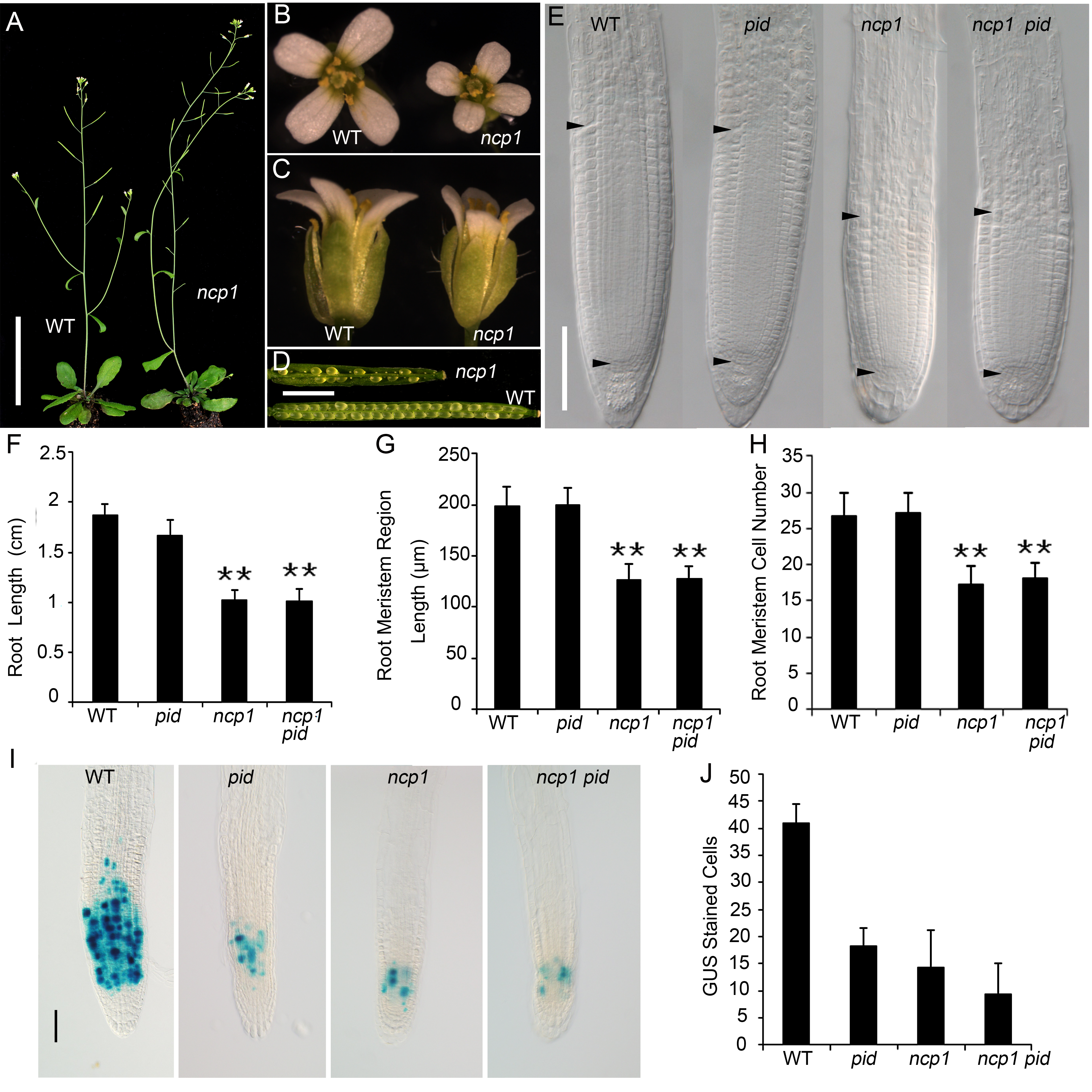

Supplement: S4 Fig — (A) The ncp1 plant is slightly taller than the WT plant. (B-E) Flowers of ncp1 are smaller (B, C) and siliques (D) are shorter with some aborted seeds, and root meristems (E) of ncp1 and ncp1 pid are shorter than WT. (F-H) Quantitative measurements of root length (F), root meristem region length (G), and root meristem cell number (H) (n = 20). (I) CycB1;1:GUS expression at 5 days after germination (DAG). (J) Quantification of CycB1;1:GUS spots (n = 10). Data are represented as mean ± SEM. Scale bar, 5 cm (A), 2 mm (D), 100 μm (E). (TIF) [file pgen.1005923.s004.tif]

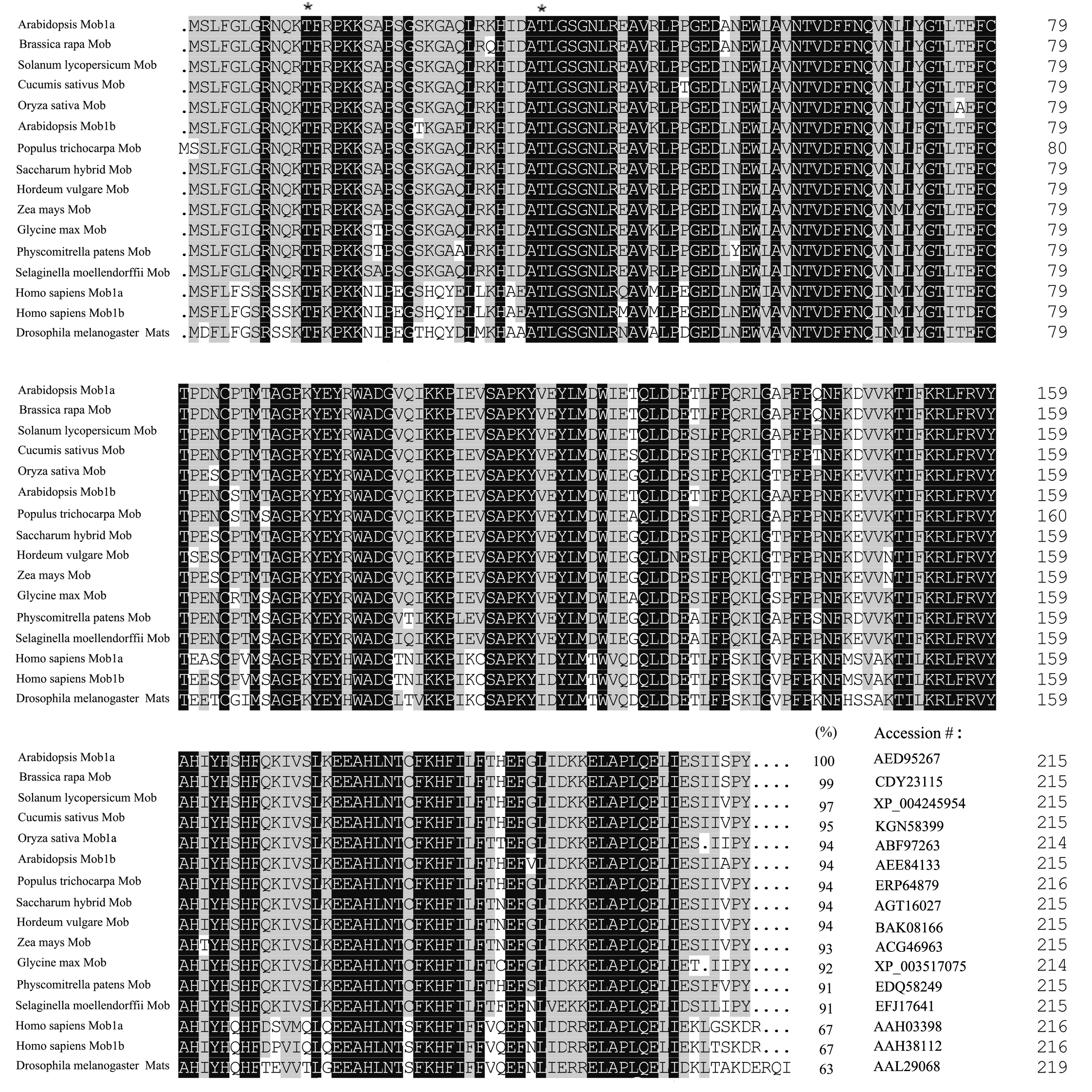

Supplement: S5 Fig — MOB1 protein sequences of representative plant and animal species are aligned. Dicotyledons: Arabidopsis thaliana, Brassica rapa, Solanum lycopersicum, Cucumis sativus, Populus trichocarpa, Glycine max. Monocotyledons: Oryza sativa, Hordeum Vulgare, Saccharum hybrid. Lycophyte: Selaginella moellendorffii. Moss: Physcomitrella patens. Mammal: Homo sapiens. Insect: Drosophila melanogaster. Percentage of identity and NCBI accession numbers are listed at the end of each sequence. Residues that are identical in all sequences aligned are highlighted in black, and similar residues are in grey. The Thr12 and Thr35 residues are labeled with asterisks. (TIF) [file pgen.1005923.s005.tif]

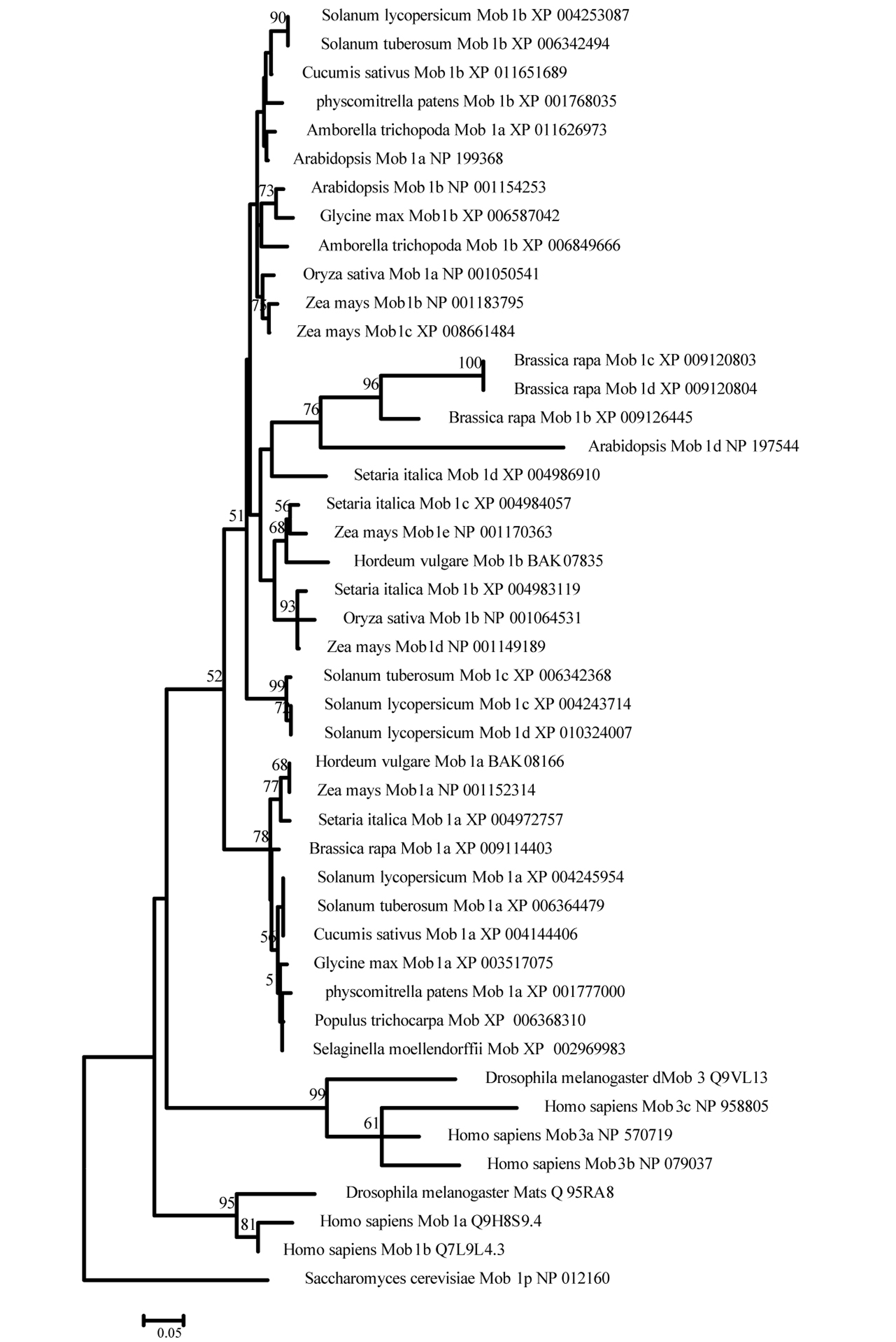

Supplement: S6 Fig — NCBI accession numbers are listed after the names of species. (TIF) [file pgen.1005923.s006.tif]

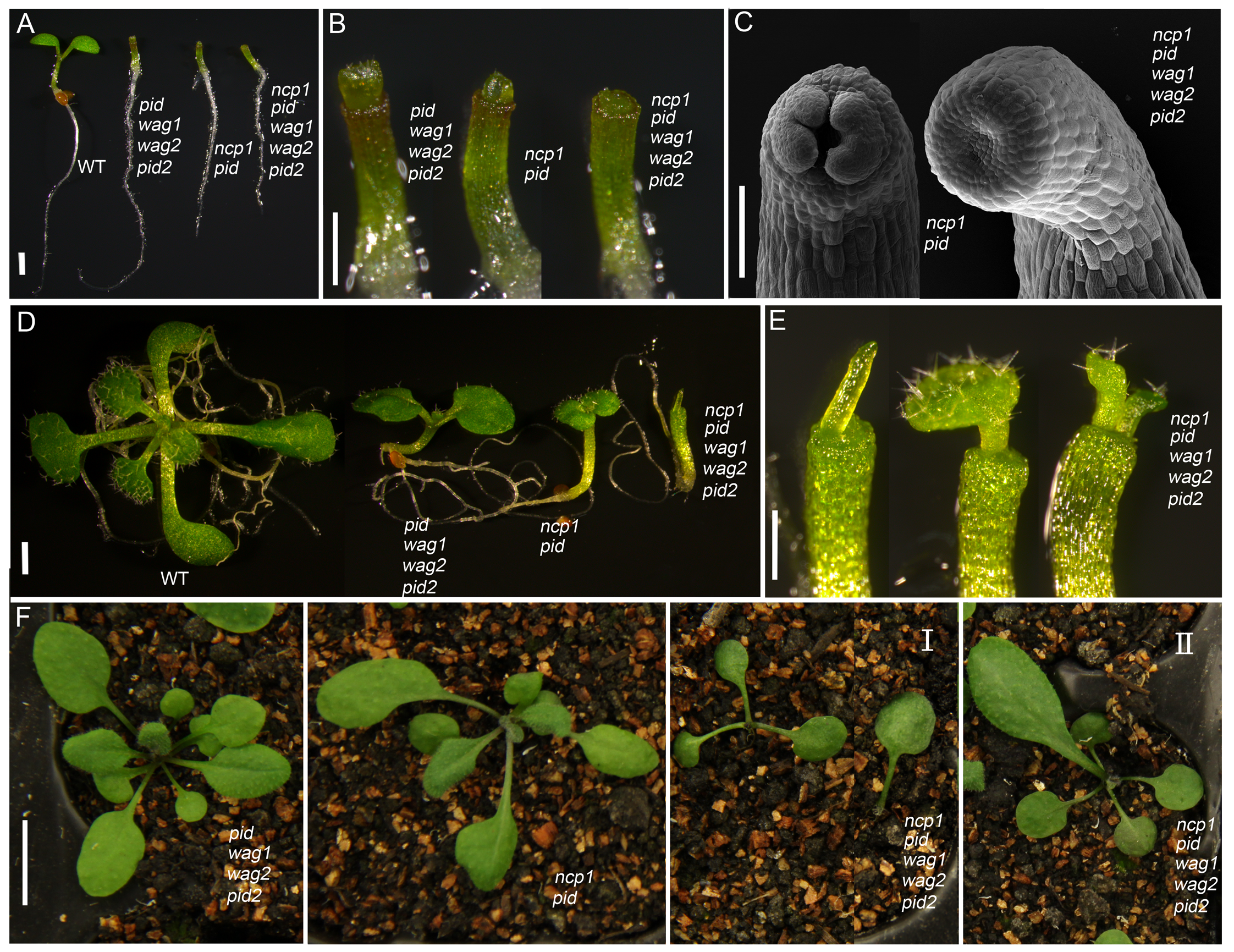

Supplement: S7 Fig — (A) Light grown seedlings at 5 DAG. (B) Close-up view of mutant seedlings in (A). (C) SEM micrograph of dark grown seedlings at 3 DAG. Note the top of the seedlings. (D) Seedlings at 14 DAG. (E) Close-up view of true leaf development in ncp1 pid wag1 wag2 pid2 mutants at 14 DAG. (F) Plants at 36 DAG. Note the type I and type II phenotypes of ncp1 pid wag1 wag2 pid2 mutants. Scale bars, 1 mm (A, D), 500 μm (B, E), 100 μm (C), 1 cm (F). (TIF) [file pgen.1005923.s007.tif]

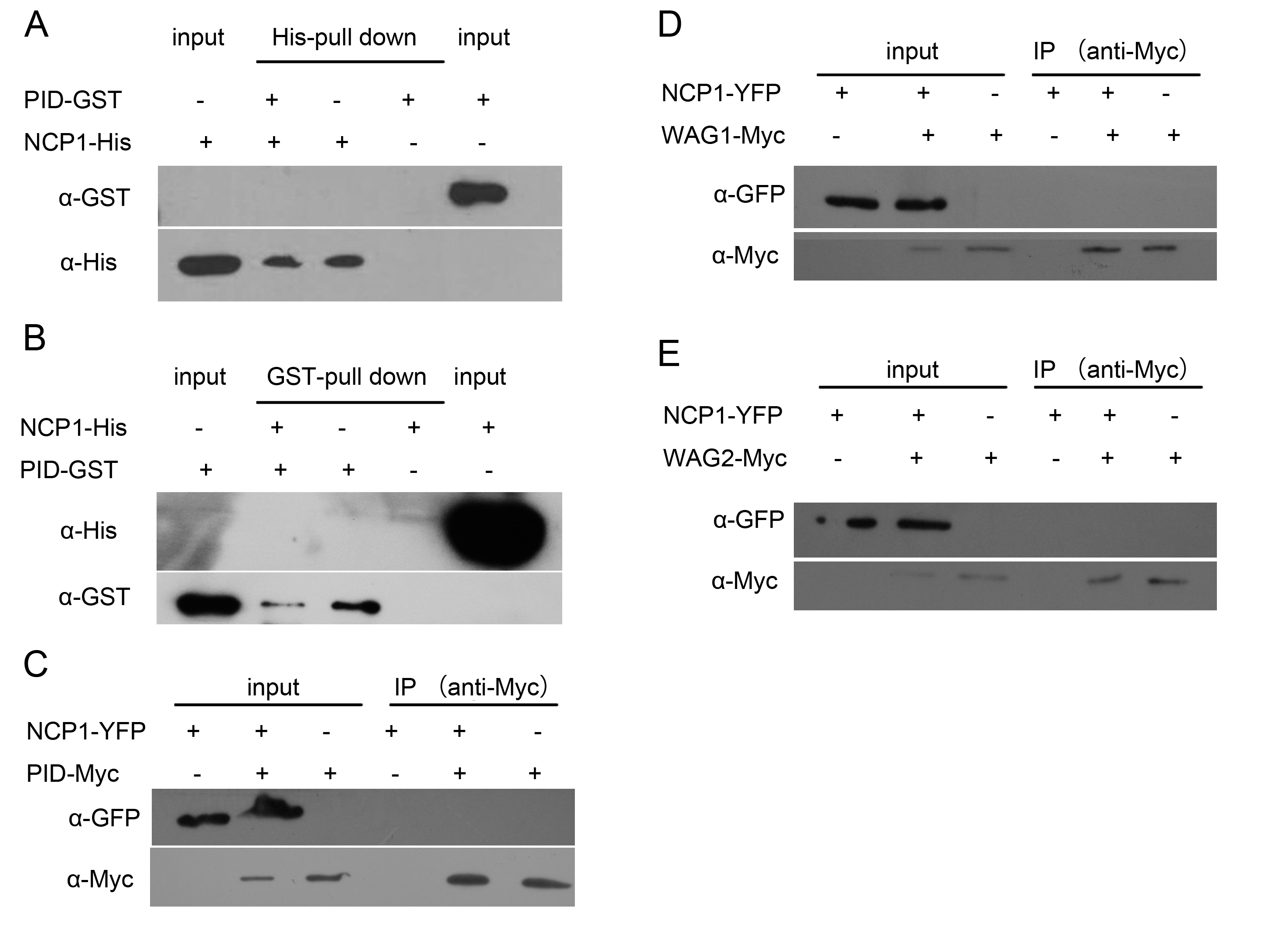

Supplement: S8 Fig — (A, B) Pull-down assay with His (A) and GST tags (B). (C-E) Co-IP assay with YFP and Myc tags. (TIF) [file pgen.1005923.s008.tif]

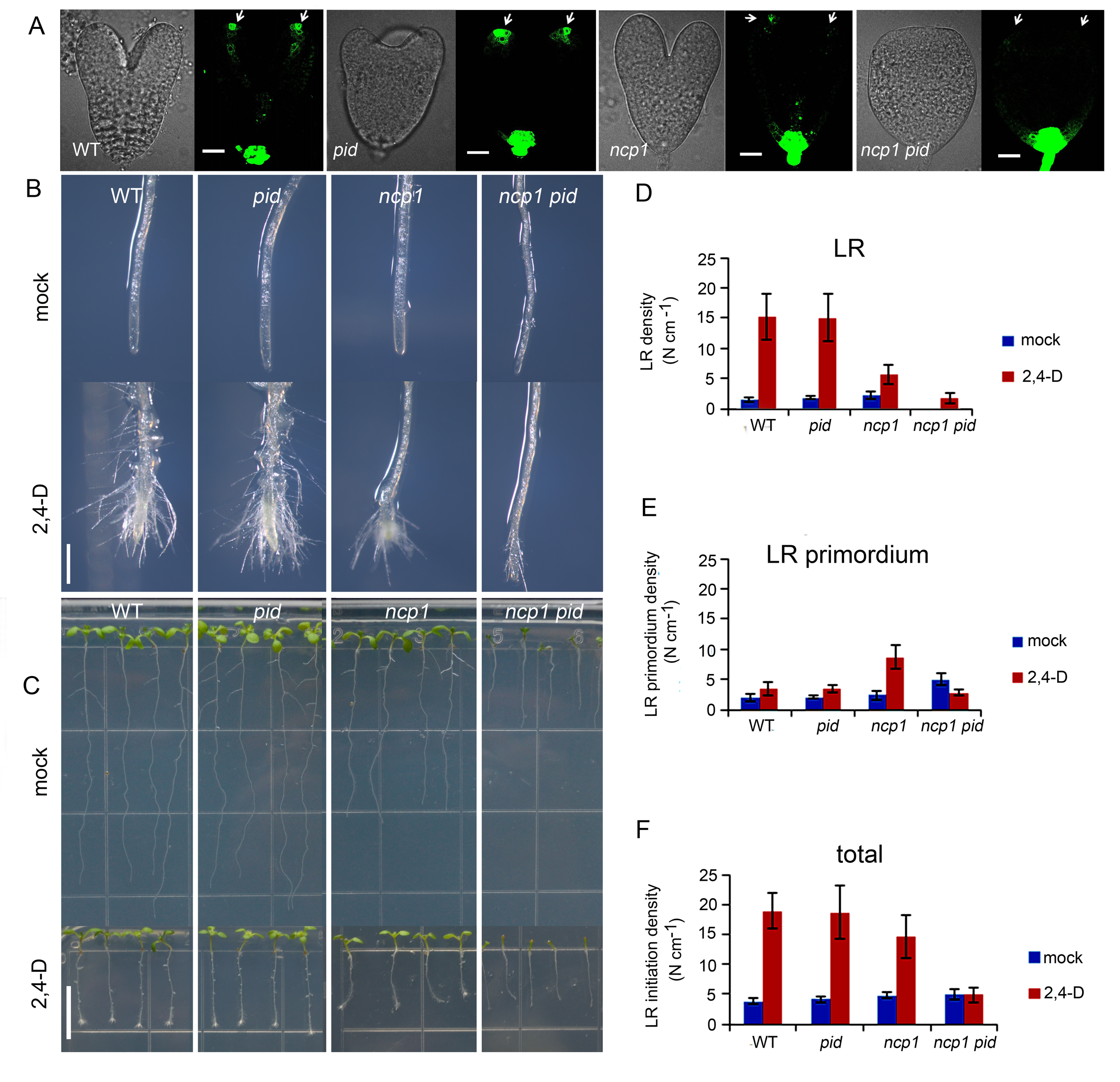

Supplement: S9 Fig — (A) DR5-GFP auxin response reporter in late heart stage embryos of WT, ncp1, pid and ncp1 pid. Note the arrowheads point to cotyledon primordia, where DR5-GFP signal was reduced in ncp1 and ncp1 pid mutants. (B) Root hair initiation and elongation of WT, ncp1, pid and ncp1 pid seedlings in response to exogenous auxin treatment. 4-day-old seedlings grown on 1/2 MS plate were transferred onto plates containing 0 nM (mock, top panel) or 50 nM 2,4-D (bottom panel) and grew for 4 days. (C) Lateral root initiation of WT, ncp1, pid and ncp1 pid seedlings in response to exogenous auxin treatment. 4-day-old seedlings grown on 1/2 MS plate were transferred onto plates containing 0 nM (mock, top panel) or 50 nM 2,4-D (bottom panel) and grew for 4 days. (D-F) Quantitative measurements of LR density (number of emerged LR per portion of the primary root where LRs are present, D), LR primordium density (number of LR primordium per portion of the primary root where LR primordia are present, E), and LR initiation density (number of non-emerged LR primordia and emerged LRs per portion of the primary root, F) of seedlings in response to exogenous auxin treatment. 4-day-old seedlings grown on 1/2 MS plate were transferred onto plates containing 0 nM (mock) or 50 nM 2,4-D and grew for 4 days. Data are represented as mean ± SEM. Scale bar, 20 μm (A), 500 μm (B), 1 cm (C). (TIF) [file pgen.1005923.s009.tif]

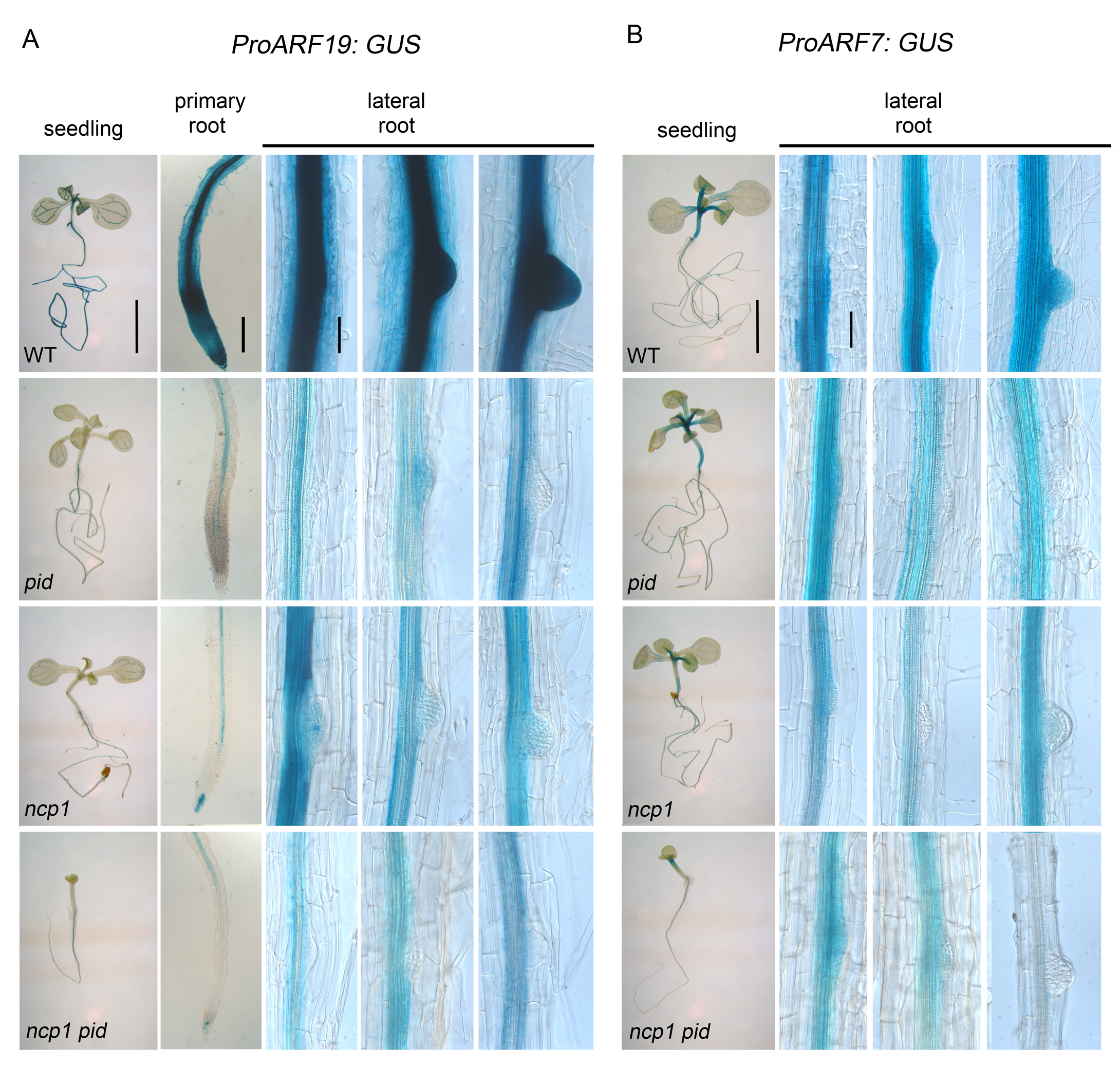

Supplement: S10 Fig — 10-d old seedlings were used for GUS staining. (A) ProARF19:GUS, from left to right: seedlings, primary roots, and lateral roots. (B) ProARF7:GUS, from left to right: seedlings and lateral roots. Scale bar, 1 mm (seedlings), 200 μm (primary roots), 50 μm (lateral roots). (TIF) [file pgen.1005923.s010.tif]

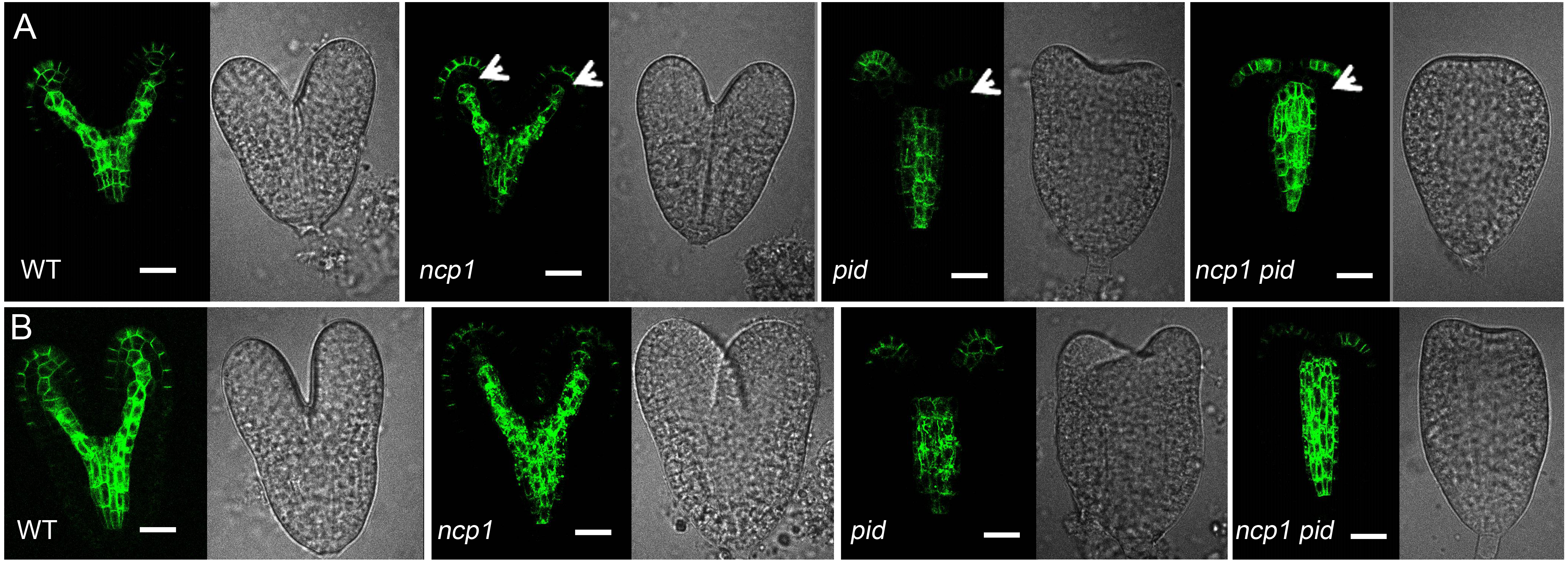

Supplement: S11 Fig — Heart (A) and late heart (B) stages of WT, ncp1, pid and ncp1 pid. Scale bar, 20 μm (A and B). Note the arrowheads point to the regions between cotyledon primordium and ground tissue, where PIN1-GFP was expressed in WT embryos but barely in mutants. (TIF) [file pgen.1005923.s011.tif]

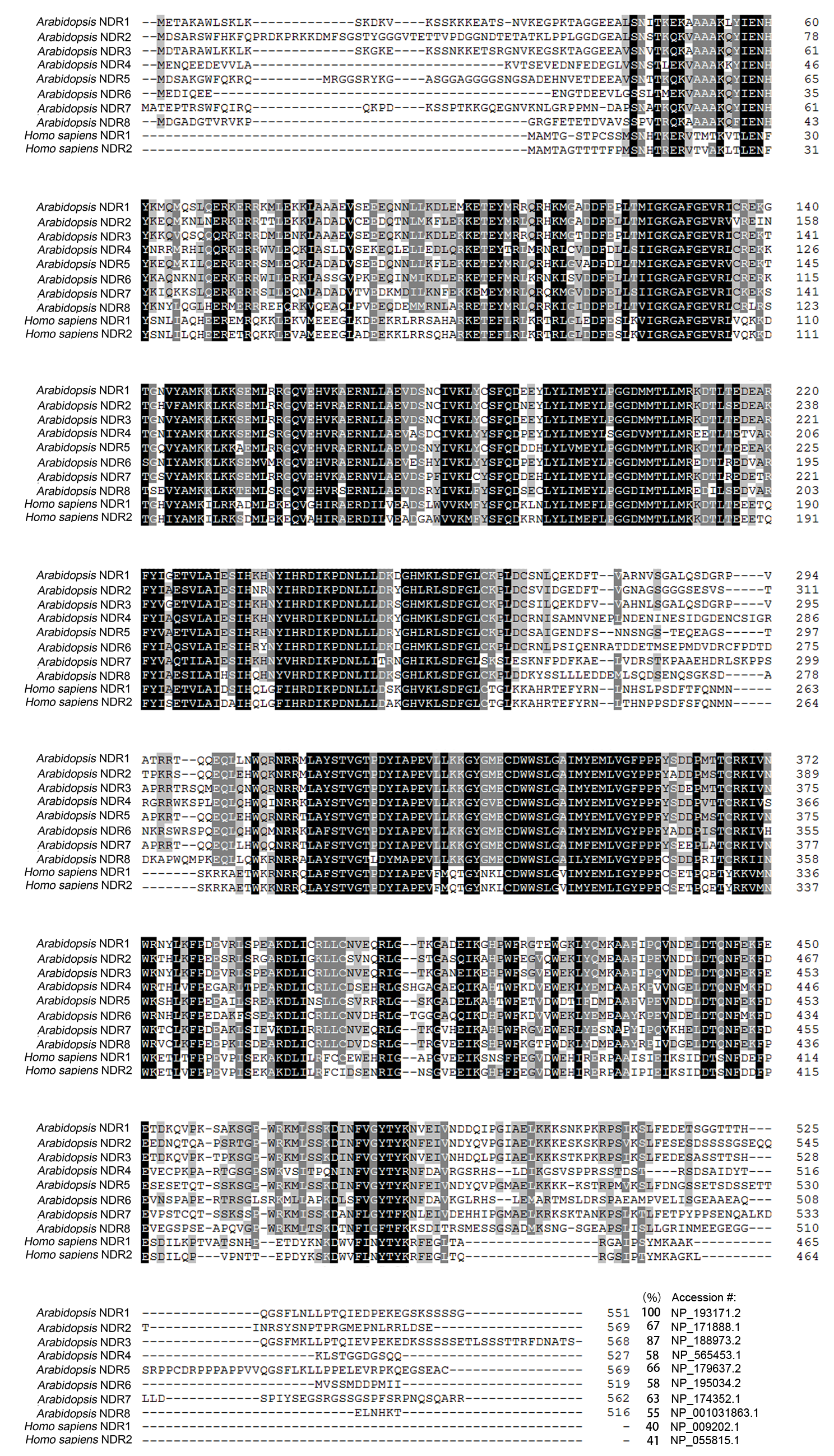

Supplement: S12 Fig — NDR/LATS protein sequences of Arabidopsis and human are aligned. (TIF) [file pgen.1005923.s012.tif]
